# Running, jumping, hunting, and scavenging: Functional analysis of vertebral mobility and backbone properties in carnivorans

**DOI:** 10.1111/joa.13955

**Published:** 2023-10-14

**Authors:** Ruslan I. Belyaev, Polina Nikolskaia, Andrey V. Bushuev, Aleksandra A. Panyutina, Darya A. Kozhanova, Natalya E. Prilepskaya

**Affiliations:** ^1^ A.N. Severtsov Institute of Ecology and Evolution Russian Academy of Sciences Moscow Russian Federation; ^2^ Geological Institute Russian Academy of Sciences Moscow Russian Federation; ^3^ Department of Vertebrate Zoology, Faculty of Biology Lomonosov Moscow State University Moscow Russian Federation; ^4^ Faculty of Life Sciences, School of Zoology Tel‐Aviv University Tel Aviv Israel; ^5^ Department of Paleontology, Faculty of Geology Lomonosov Moscow State University Moscow Russian Federation

**Keywords:** Canidae, Carnivora, Felidae, gallop, hunting strategies, regionalization, vertebral biomechanics

## Abstract

Carnivorans are well‐known for their exceptional backbone mobility, which enables them to excel in fast running and long jumping, leading to them being among the most successful predators amongst terrestrial mammals. This study presents the first large‐scale analysis of mobility throughout the presacral region of the vertebral column in carnivorans. The study covers representatives of 6 families, 24 genera and 34 species. We utilized a previously developed osteometry‐based method to calculate available range of motion, quantifying all three directions of intervertebral mobility: sagittal bending (SB), lateral bending (LB), and axial rotation (AR). We observed a strong phylogenetic signal in the structural basis of the vertebral column (vertebral and joint formulae, length proportions of the backbone modules) and an insignificant phylogenetic signal in most characteristics of intervertebral mobility. This indicates that within the existing structure (stabilization of which occurred rather early in different phylogenetic lineages), intervertebral mobility in carnivorans is quite flexible. Our findings reveal that hyenas and canids, which use their jaws to seize prey, are characterized by a noticeably elongated cervical region and significantly higher SB and LB mobility of the cervical joints compared to other carnivorans. In representatives of other carnivoran families, the cervical region is very short, but the flexibility of the neck (both SB and LB) is significantly higher than that of short‐necked odd‐toed and even‐toed ungulates. The lumbar region of the backbone in carnivorans is dorsomobile in the sagittal plane, being on average ~23° more mobile than in artiodactyls and ~38° more mobile than in perissodactyls. However, despite the general dorsomobility, only some representatives of Canidae, Felidae, and Viverridae are superior in lumbar flexibility to the most dorsomobile ungulates. The most dorsomobile artiodactyls are equal or even superior to carnivorans in their ability to engage in dorsal extension during galloping. In contrast, carnivorans are far superior to ungulates in their ability to engage ventral flexion. The cumulative SB in the lumbar region in carnivorans largely depends on the mode of running and hunting. Thus, adaptation to prolonged and enduring pursuit of prey in hyenas is accompanied by markedly reduced SB flexibility in the lumbar region. A more dorsostable run is also a characteristic of the Ursidae, and the peculiar maned wolf. Representatives of Felidae and Canidae have significantly more available SB mobility in the lumbar region. However, they fully engage it only occasionally at key moments of the hunt associated with the direct capture of the prey or when running in a straight line at maximum speed.

## Graphical abstract

Intervertebral mobility and backbone properties were studied in 34 species of terrestrial carnivorans. Carnivorans that seize prey with their jaws (canids and hyaenids) are characterized by a significantly elongated neck and increased vertebral mobility in the sagittal and horizontal planes. The lumbar spine in carnivorans is on average more mobile in the sagittal plane than in artiodactyls and perissodactyls. However, ungulates are on average more dorsomobile in regard to axial rotation in the thoracic region.

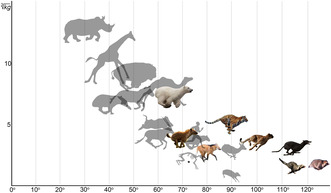

## 1 INTRODUCTION

Modern‐day representatives of the order Carnivora are the most successful and impressive predators in the terrestrial fauna of amniotes. Carnivorans occupy various ecological niches, adapting to diverse food sources, with their sizes ranging from small mustelids weighing a few hundred grams to bears and big cats weighing several hundred kilos. Carnivorans are renowned for their running abilities, including speed, stamina, and maneuverability, which are retained across a wide range of body sizes. These features occur in different combinations in various carnivorans and are closely related to their foraging strategies.

Gambaryan (1974) noted that the mode of hunting is a key factor determining the biomechanics of running in various carnivorans. Foraging strategies in carnivorans are extremely diverse and include adaptations to prolonged pursuit of prey, rapid pursuit at short distances, sneaking and jumping, collective and individual hunting, fishing, underwater hunting, hunting or foraging in tree branches, hunting in burrows, durophagy, scavenging. Some carnivorans transition to omnivory, or even frugivorous/folivorous diets (Wilson & Mittermeier, 2009). Adaptations to a particular foraging strategy determine the locomotor mode, which is linked to the function of the vertebral column and its mobility. In addition to differences in hunting strategies, carnivorans also differ in their prey‐catching strategies. Thus, Felidae mainly use their paws to seize their prey, while Canidae and Hyaenidae use their jaws (Nowak, 2005).

This study examines the mobility of the presacral vertebral column in terrestrial species of Carnivora. The vertebral column of carnivorans is well known for its mobility. The difference in the backbone flexibility is so pronounced that Gambaryan (1974) classified ungulates and carnivorans that compete in running as predators and prey as “the dorsostable runners” and “the dorsomobile runners,” respectively. Hildebrand (1959) used the horse and cheetah as model objects that characterize opposite adaptations to galloping. Contrary to this opposition, Alexander et al. (1985) suggested the positive role of backbone elasticity in galloping at high speeds for both carnivorans (domestic dog) and artiodactyls (fallow deer).

Previous studies of vertebral column biomechanics in carnivorans have been almost exclusively focused on the lumbosacral part of the backbone, which is actively involved in mammalian asymmetric gaits (Schilling & Carrier, 2010). Two different approaches have been used to study Range of Motion (ROM) in carnivorans. One approach is the measurement of available ROM (aROM) or ‘mobility’ on syndesmological specimens of vertebral columns (so called in vitro studies). The other approach is the measurement of used ROM (uROM) or ‘movement’ in living animals (in vivo studies). Only in a few studies, all three directions of intervertebral movement or mobility have been analyzed: sagittal bending (SB), lateral bending (LB), and axial rotation (AR).

So‐called in vitro (actually ex vivo) studies are based on the study of specially prepared syndesmological specimens, cleared of muscles but retaining ligaments. In early in vitro studies (Gál, 1993; Pylypchuk, 1975), the aROM values of the specimens were estimated using X‐ray. The lumbosacral region was bent as a whole, not segmented into functional spinal units (FSU – pairs or triples of vertebrae used in such experiments today). Pylypchuk (1975) studied SB and LB aROM in the lumbar and lumbosacral joints of an unspecified breed of dog (*Canis lupus familiaris*; *n* = 3). Gál (1993) studied SB aROM in the lumbar and lumbosacral joints of the tiger (*Panthera tigris*; *n* = 1), jaguar (*Panthera onca*; *n* = 1), badger (*Meles meles; n =* 2), and harbor seal (*Phoca vitulina*; *n* = 1). In a study by Benninger et al. (2004), a special spine tester was used to measure amplitudes of intervertebral motion. As a result, all three directions of the aROM (SB, LB, AR) at the L4‐S1 locus in dogs (*n* = 25) were studied. The sample studied by Benninger et al. (2004) included eleven different breeds of dogs (mostly German Shepherds; *n* = 9). It is worth noting that the L4‐S1 locus was studied by Benninger et al. (2004) without segmentation, and the applied moment (torque) was lower than in artiodactyls (M = ±3 Nm vs. ±7.5 Nm, respectively; Wilke et al., 1997a,b, 2011). Finally, a recent study by Jones et al. (2020) examined all three directions of aROM in the entire presacral region of the vertebral column in the domestic cat (*Felis catus*; *n* = 3–5 per joint).

In vivo studies are extremely complex and require researchers to use special equipment for sufficiently accurate fixation of uROM amplitudes during lifetime activities. Early studies employed a highly invasive method using bone implants (Steinmann pins) instrumented with spatial linkages to measure uROM between adjacent vertebrae (Buttermann et al., 1992; Wood et al., 1992). In these papers, uROM and loads in the zygapophysial facet joint for a single joint (L2‐L3) of large (25–30 kg) mongrel dogs were studied. Such methods have been replaced by kinematic analysis of markers fixed on the skin while animals are walking (Gradner et al., 2007). In this study, markers were fixed only on certain selected vertebrae of the cervical, thoracic, lumbar, and sacral spine allowing evaluation of all three directions of uROM (SB, LB, AR), for regional vertebral kinematics. The most accurate and complete uROM values were obtained in a study using an X‐ray rotoscoping technique (combining biplanar high‐speed X‐ray video and the reconstruction of skeletal elements from CT bone scans). These techniques allowed the study of all three directions of uROM (SB, LB, and AR) in each of the studied intervertebral joints. Using these techniques, uROM data were obtained from the L1‐S1 joints in walking and trotting beagles (Wachs et al., 2016). In large mammals (horses and dogs), in vivo studies of vertebral column movements are usually carried out on a treadmill, which greatly limits the animal's ability to display the full uROM in all three directions. For example, dogs only use about 1/10th of the SB aROM in the lumbosacral joint during walking and trotting (3–4° vs. 28–37°; Benninger et al., 2004; Pylypchuk, 1975; Wachs et al., 2016). The cumulative SB uROM during gallop in carnivorans has been only roughly estimated in greyhounds by the general curvature of the vertebral column as a whole (Alexander et al., 1985; Muybridge, 1887).

The study of shape, regionalization, and modularity has been an important area of research on the carnivoran spine in recent years (Martín‐Serra et al., 2021; Randau & Goswami, 2018). These studies show that the vertebral column in carnivorans is well regionalized into three sets of vertebrae (cervical, anterodorsal, and posterodorsal) with one transitional (diaphragmatic) vertebra between the antero‐ and posterodorsal modules. The clear modular structure of the backbone indicates the functional regionalization along the vertebral column, which led to the module formation. However, the vertebral column mobility of carnivorans has never been studied quantitatively across a wide range of species.

The purpose of this study is to conduct the first large‐scale analysis of the three directions of mobility throughout the presacral region of the vertebral column (excluding atlas‐axis and lumbosacral joint) and some backbone properties in terrestrial carnivorans. Among other questions in this study, we want to assess the following: (1) the regionalization of the vertebral column in six studied families of Carnivora; (2) whether the mobility of the neck depends on the method of capturing the prey (with the jaws or paws); (3) whether it is possible to distinguish in carnivorans studied a relationship between the lumbar SB aROM and prevalent running and/or hunting techniques; (4) whether carnivorans are able to engage full SB aROM of the backbone while galloping; (5) whether the gallop of carnivorans is actually more dorsomobile than the gallop of ungulates.

## 2 MATERIAL

In this study, we utilized dry osteological material from museum collections, namely the Musée National d'Histoire Naturelle du Luxembourg (MNHN), Luxembourg; Zoological Institute of the Russian Academy of Sciences (ZIN), Saint Petersburg; and the Zoological Museum of the Lomonosov Moscow State University (ZMMU), Moscow. The study covered representatives of 6 extant terrestrial Carnivora families, encompassing 24 genera, 34 species (56 specimens, Table S1). In eight specimens belonging to five species of small mustelids, only vertebral and joint formulae, as well as length proportions of the backbone modules were studied. Also, one specimen of pine marten (*Martes martes* PAN‐160) from A.N. Severtsov Institute of Ecology and Evolution of the Russian Academy of Sciences (IEE) was studied with X‐rays. For comparison, we included a dataset of 39 species (53 specimens) of artiodactyls and 15 species (29 specimens) of perissodactyls, whose mobility in the entire presacral region of the vertebral column had been previously studied (Belyaev et al., 2021b, 2023; Table S1). Juvenile specimens were excluded from the analysis, as they display distinct differences in vertebral morphology compared to adults (Benninger et al., 2004). Specimens with unfused end‐plates to the vertebral bodies were considered juvenile. Some of the studied carnivorans were animals kept in captivity. Life in captivity can lead to an increased pathologies, including backbone pathologies, and changes in bone morphology compared with animals living in the wild (Canington et al., 2018; O'Regan & Kitchener, 2005). Thus, the results obtained here may vary to some extent from samples of the same species from the wild.

### 2.1 Terminology for vertebral column parts

**FIGURE 1:**
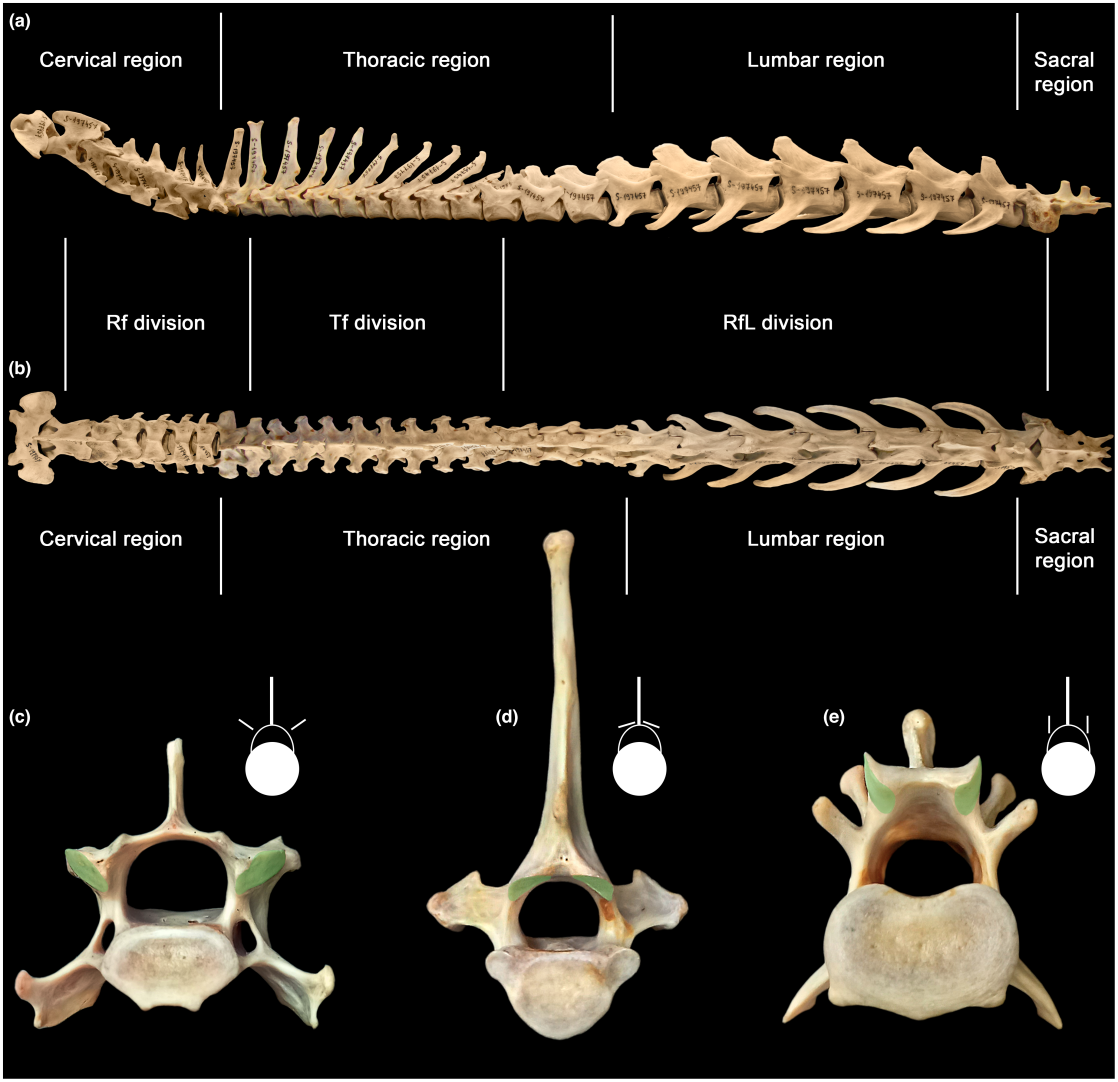
Regions (cervical, thoracic, lumbar, and sacral) and divisions (radial [Rf], tangential [Tf] and radial with a lock [RfL] facet types) of the vertebral column exemplified by *Lynx lynx* (ZMMU S‐197457). Left lateral (a) and dorsal (b) view of the entire vertebral column. (c)–(e) posterior view of selected vertebrae showing different orientations of postzygapophysial articular facets: Rf in C4 (c), Tf in T3 (d), RfL in L1 (e).

The regions (cervical, thoracic, lumbar, sacral) are traditionally defined by rib features: cervical ribs are pleurapophyses, thoracic ribs are free, lumbar ribs are absent or fused to transverse processes (Filler, 2007), and sacral ribs mediate sacroiliac fusion (Figure 1). Neck mobility refers to intracervical joints posterior to the axis (C2‐C7). Thoracic region mobility refers to intrathoracic joints from T1‐T2 up to the joint between the penultimate and ultimate thoracic vertebrae (Table S1). Lumbar region mobility refers to intralumbar joints from L1 to L2 up to the joint between the penultimate and ultimate lumbar vertebrae. Lumbosacral mobility refers to all the intralumbar joints plus the lumbosacral (LS) one. In this study, we assigned the neck‐thorax joint (C7‐T1) to the cervical region and the joint located between the last thoracic vertebra and L1 to the lumbar region. Thus, referring to the thoracic region, we only mean intrathoracic joints. SB aROM in the lumbosacral joint was not studied due to the reasons explained in Section 3.2.

Divisions are distinguished by zygapophysial facet type. There is a succession of radial facets (Rf), tangential facets (Tf), and radial facets with a lock (RfL) from the anterior end to the posterior end of the presacral vertebral column (Figure 1c–f). V‐like (Figure 1c) and ‐like (Figure 1e) patterns were named “radial facets” and “tangential facets” by Virchow (1907). The U‐like pattern (Figure 1f) was named “radial facets with a lock” by Kuznetsov and Tereschenko (2010). The abbreviations Rf, Tf, and RfL, respectively, were introduced by Belyaev et al. (2021a). Transitional vertebra with Rf prezygapophyses and Tf postzygapophyses are usually T2, sometimes T1. Transitional vertebra with Tf prezygapophyses and RfL postzygapophyses, historically termed “diaphragmatic vertebra” (Slijper, 1946), vary from T9 to T13 (Table S1). In Felidae and Canidae, diaphragmatic vertebra always correspond to weakly‐integrated transitional vertebrae (in terms of Martín‐Serra et al., 2021) between the anterodorsal and posterodorsal modules. In other carnivoran families, the morphological differences between vertebrae in modules are visually much less pronounced.

Our RfL division corresponds to the posterodorsal module of the backbone (Martín‐Serra et al., 2021) plus the lumbosacral joint and the joint between the diaphragmatic vertebra and the posterodorsal module. Our Tf division is notably shorter than the anterodorsal module of the backbone (Martín‐Serra et al., 2021), which is integrated with both C6 and C7. Tf joints in carnivorans usually begin from T2–T3 joint.

## 3 METHOD

### 3.1 Amplitude calculations

To calculate intervertebral mobility, we employed a recently established osteometry‐based method (Belyaev et al., 2021a). This approach is based on the assumption of the functional interrelation between aROM and the geometry of vertebrae and, in particular, of zygapophysial articular facets (Kuznetsov & Tereschenko, 2010).

In this study, we calculated the amplitudes of motion (aROM values) for three degrees of freedom in every presacral intervertebral joint, except the atlas‐axis (number 1) and lumbosacral joint. For SB, the calculated amplitude of motion is the sum of ventral flexion and dorsal extension; for LB and AR, it is the sum of respective motions to the left and right. We analysed both the mean amplitudes of motion in the intervertebral joints and the cumulative aROM values in different regions and divisions of the vertebral column.

The approach used was designed to derive aROM estimates from dry vertebral osteometry with the help of a pair of adjustable coefficients (*K*_S_ and *K*_R_). Their values were adjusted (Belyaev et al., 2021a) to match the estimate to direct in vitro aROM measurements on syndesmological preparations of vertebral columns of human (Wen et al., 1993; Wilke et al., 2017; Yamamoto et al., 1989) and a few reference species of artiodactyls (sheep, pig, and cow; Wilke et al., 1997a,b, 2011). Thus, the calibrated formulae allow the calculation of syndesmological aROMs based on vertebrae osteometry. The optimal values of coefficients *K*_S_ and *K*_R_ were found by minimizing the standard deviation (SD) between the (presumably true) reference and calculated values of aROM using the Microsoft Excel Solver Add‐In tool (Microsoft Corporation). The Solver tool is used for calculations by formulae in which variables are present. The Solver tool selects the optimal values of the variables (in our case *K*_S_ and *K*_R_ values) for best matching of the calculation results to the selected criterion.

Trigonometric formulae were used for aROM calculation (Table 1). The approach is based on the idea that articular surfaces resemble segments of surfaces of rotation (Figure S2.1). Therefore, an angle of rotation in a chosen projection can be calculated (in radians) by dividing the length of the arc of rotation by the radius of this arc. The length of the arc of rotation is calculated from the difference in lengths of the arcs of two articulating surfaces and their maximum non‐overlap constraint introduced with a coefficient *K*_S_. More specifically, *K*_S_ determines the fraction of the smaller facet which slides out of the larger one in the marginal positions of joint motions (Figure S2.3a–c). The second coefficient *K*_R_ adjusts the radius of rotation in the joint (Figure S2.3d–f).

**TABLE 1:** Formulae for aROM calculations used in this study.

Motion type	Facet type	Formula	*K*_R_	*K*_S_
SB	Rf	φ = [arcsin(*L*_long_/**1.38** *R*_vert_) – **0.72**arcsin(*L*_short_/**1.38** *R*_vert_)]•360/π	0.69	0.14
	Tf	φ = [arcsin(*L*_long_/**2** *R*_vert_) – **0.76**arcsin(*L*_short_/**2** *R*_vert_)]•360/π	1	0.12
	RfL	φ = [arcsin(*L*_long_/**2** *R*_vert_) – **0.46**arcsin(*L*_short_/**2** *R*_vert_)]•360/π	1	0.27
LB	Rf	φ = [arcsin(*L*_long_/**2** *R*_lat_) – **0.18**arcsin(*L*_short_/**2** *R*_lat_)]•360/π	1	0.41
	Tf	φ = [arcsin(*W*_long_/**2** *R*_lat_) – **0.52**arcsin(*W*_short_/**2** *R*_lat_)]•360/π	1	0.24
	RfL	φ = [arcsin(*D*_max_long_/**2** *R*_lat_) – **0.86**arcsin(*D*_max_short_/**2** *R*_lat_)]•360/π	1	0.07
AR	Rf	φ = [arcsin(*W*_long_/**2** *R*_vert_) – **0.74**arcsin(*W*_short_/**2** *R*_vert_)]•360/π	1	0.13
	Tf	φ = [arcsin(*W*_long_/**2** *R*_lat_) – **0.54**arcsin(*W*_short_/**2** *R*_lat_)]•360/π	1	0.23
	RfL	φ = [arcsin(*D*_max_long_/**2** *R*_lat_) – arcsin(*D*_max_short_/**2** *R*_lat_)]•360/π	1	0

*Note*: In the RfL division formulae was optimized for carnivores. Optimized coefficient values are specified in the right columns.

During the validation of the optimal formulae for calculating aROM values, it was found that separate formulae for different zygapophysial facet types (Rf, Tf, RfL) give significantly greater accuracy in aROM calculation than the formulae for the presacral vertebral column as a whole, and greater accuracy than the separate formulae for different vertebral column regions (cervical, thoracic, lumbar; Belyaev et al., 2021a). Thus, the *K*_S_ and *K*_R_ coefficients are zygapophysial facet‐specific (Rf, Tf, and RfL) and mobility‐specific (SB, LB, and AR), resulting in 3 × 3 = 9 optimal specific values of each coefficient for the whole vertebral column. The measurements used (Figure S2.2) were shown and explained in detail by Belyaev et al. (2021a); Figure 1 and Figure S2 therein). A workflow illustrating how aROM values are calculated can be found in Tables S1–S4 in Belyaev et al. (2021a).

As accuracy of measurements is crucial for the method used in this study, we calculated the mobility of the backbone only in the largest representatives of Mustelidae. However, as typical mustelids are small‐sized and very agile predators, we also conducted further characterization of available SB aROM of the backbone using X‐rays. For the analysis, we used the carcass of a pine marten (*Martes martes* PAN‐160) from the institute's collection, which was preliminarily defrosted before being imaged. X‐ray images of the specimen were taken in the lateral view in three poses: with its backbone fully dorsally extended and with its backbone ventrally flexed in two different ways. Images were acquired at 55 kV, 0.1 mA, 2 s with Multi‐Purpose Mobile X‐Ray Diagnostic Installation PRDU (“ELTEH‐Med” company).

### 3.2 Formulae optimization for carnivorans

The approach used allows any researcher to solve new optimal *K*_S_ and *K*_R_ values, adapting the formulae to a specific group of mammals. The availability of reliable in vitro reference data for the lumbar region in various carnivorans allowed us to calculate new optimal values for the *K*_R_ and *K*_S_ coefficients used in the formulae for RfL division of the vertebral column. We used all reliable data on intervertebral mobility for this purpose, including studies of SB in the L1‐S1 joints in the domestic dog (Pylypchuk, 1975, *n* = 3), jaguar, and tiger (Gál, 1993, *n* = 1), as well as studies of all three directions of intervertebral mobility (SB, LB, AR) at L4‐S1 joints in dog (Benninger et al., 2004, *n* = 25) and L1‐S1 in domestic cat (Jones et al., 2020, *n* from 3 to 5 in different joints). All studied individuals of the dog (including wolves, *n* = 5), cat (*n* = 2), jaguar and tiger (*n* = 4) were used to solve optimal pair of the *K*_R_ and *K*_S_ coefficients; we used our dogs+wolves subsample twice, separately for the Pylypchuk (1975) and Benninger et al. (2004) data.

Since each species of carnivoran had a different number of joints studied, we used the lowest average SD between calculated aROM and the reference in vitro aROM values in four subsets, i.e., (SD *Canis* (Pylypchuk, 1975) + SD *Canis* (Benninger et al., 2004) + SD *Felis* + SD *Panthera*)/4 for SB and (SD *Canis* (Benninger et al., 2004) + SD *Felis*)/2 for LB and AR, as the optimal criterion. This ensured a fair comparison across species, avoiding distortion in favor of those for which a greater number of joints were studied. This distortion would occur if optimization were carried out by minimizing overall SD, i.e., SD (*Canis* + *Canis* + *Felis* + *Panthera*).

For other divisions of the vertebral column (Rf and Tf), where reliable in vitro reference data for calibration were lacking, we used the same coefficients calibrated with in vitro data of sheep, pig, and cow (Wilke et al., 1997a,b, 2011), as used in previous studies on odd‐toed and even‐toed ungulates (Belyaev et al., 2021b, 2023). This ensures that all the intergroup/interspecific differences in aROMs subsequently identified in this study are associated with the geometry of the vertebrae only. The modified SB formula (Belyaev et al., 2022) for the lumbosacral joint is not suitable for carnivorans due to their lack of a well‐pronounced prezygapophysial postfacet fossa with stopper on the first sacral vertebra. Consequently, we refrained from calculating mobility in the LS joint, which is characterized by SB‐hypermobility in carnivorans (Benninger et al., 2004; Gál, 1993; Jones et al., 2020; Pylypchuk, 1975); calculation of SB aROM using formula for typical RfL joints would lead to a serious underestimation of the actual amplitude of motion.

### 3.3 Length measurements

To study the relationship between the linear dimensions of the vertebral column and intervertebral mobility, we measured the lengths of the vertebral regions (cervical, thoracic, lumbar, sacral) and divisions (Rf, Tf, RfL; Table S1). The length was measured on the articulated vertebral columns, along the ventral sagittal line. For example, the length of the cervical region was measured from the anterior edge of C1 (atlas) to the posterior edge of C7, etc. These ‘osteological lengths’ underestimate the actual length by the total length of the intervertebral disks in the respective region or division.

Based on these measurements, we calculated the following proportions: cervical region length/trunk length (the latter is thoracolumbar plus sacral length); thoracic region length/thoracolumbar length; lumbar region length/thoracolumbar length; Tf division length/thoracolumbar length; RfL division length/thoracolumbar length.

### 3.4 Data analysis

The data analysis was performed using IBM SPSS Statistics 23, taking into account the model limitations described in detail in previous studies (Belyaev et al., 2021a, 2021b). Data from carnivorans (*n* = 56) were analyzed alongside a previously published dataset of even‐toed ungulates (*n* = 53) and odd‐toed ungulates (*n* = 29).

Before conducting the analysis, the variables were tested for normality using the Kolmogorov–Smirnov (*K*‐*S*) test. The *K*‐*S* test results are shown in Table 2 and Tables S4.1‐S4.4. If the distribution was found to be normal, parametric statistics were employed for further analysis. Student's *t*‐test (independent samples) was used to compare the mobility in carnivorans versus artiodactyls. Analysis of variance (ANOVA) was used to compare the mobility in different regions and divisions of the vertebral column among various groups of carnivorans and ungulates. On the other hand, if the *K*‐*S* test indicated a non‐normal distribution, non‐parametric statistics were employed. So, the Kruskal–Wallis *H* test was used to compare the mobility in different regions and divisions of the vertebral column.

**TABLE 2:** Numerical characteristics and length proportions of the vertebral column in carnivorans.

Variable	*N*	Min	Max	Mean	SD	*K*‐*S* test (*p*)	Family		Species	
							Min	Max	Min	Max
No. C + T + L vertebrae	56	26	28	26.93	0.38	<0.001	Multiple	Multiple	Multiple	Multiple
No. T vertebrae	56	12	15	13.52	0.76	<0.001	Multiple	Multiple	Multiple	Multiple
No. L vertebrae	56	4	7	6.41	0.78	<0.001	Hyaenidae	Multiple	*Crocuta crocuta*	Multiple
No. S vertebrae	54	2	6	3.37	0.94	<0.001	Viverridae	Ursidae	Multiple	Multiple
No. Rf joints	56	6	7	6.96	0.19	<0.001	Multiple	Multiple	Multiple	Multiple
No. Tf joints	56	7	11	8.80	0.96	<0.001	Multiple	Hyaenidae	Multiple	Multiple
No. RfL joints	56	8	12	10.16	0.97	<0.001	Hyaenidae	Canidae	Multiple	*Canis familiaris*
Length ratio C/(T + L + S) (%)	55	17.6	52.8	31.4	7.4	<0.001	Mustelidae	Hyaenidae	*Enhydra lutris*	*Hyaena hyaena*
Length ratio T/(T + L) (%)	56	47.9	74.8	59.1	6.0	0.200	Felidae	Hyaenidae	*Acinonyx jubatus*	*Crocuta crocuta*
Length ratio L/(T + L) (%)	56	25.2	52.1	40.9	6.0	0.200	Hyaenidae	Felidae	*Crocuta crocuta*	*Acinonyx jubatus*
Length ratio Tf/(T + L) (%)	56	29.6	58.9	40.8	6.1	0.045	Viverridae	Hyaenidae	*Acinonyx jubatus*	*Crocuta crocuta*
Length ratio RfL/(T + L) (%)	56	35.9	67.0	55.4	6.3	0.010	Hyaenidae	Viverridae	*Crocuta crocuta*	*Acinonyx jubatus*

Abbreviations: C, cervical; L, lumbar; S, sacral; T, thoracic.

*Note*: *N* – number of skeletons involved in each measurement.

Alongside one‐way ANOVA, three methods of post hoc multiple comparisons were utilized: Duncan, Scheffe, and Tukey's HSD. To control the family‐wise error rate, the Holm–Bonferroni method was applied. The taxonomic grouping (orders and families) served as the grouping variable for ANOVA.

For all phylogenetic comparative analyses, R v4.3.1 (R Core Team, 2023) was used. The rooted MCC consensus DNA‐only phylogenetic tree was obtained from the VertLife project (Upham et al., 2019). To make the tree ultrametric, the ‘chronos’ function from the ‘ape’ package (Paradis et al., 2004; Paradis & Schliep, 2019) was applied, with the parameters (lambda = 0, model = ‘discrete’) chosen based on the Phylogenetic Information Criterion (ΦIC) scores (Paradis, 2013). The calibration argument, representing the minimum and maximum age of the root node (52.7–46.7 Mya, the age when crown Carnivora divided into Caniformia and Feliformia), was taken from Hassanin et al. (2021).

To account for phylogenetic non‐independence among species means in the principal component analysis (PCA), a phylogenetic PCA (pPCA; Jombart et al., 2010; Polly et al., 2013; Revell, 2009) was conducted using the ‘phyl.pca’ function from the ‘phytools’ package (Revell, 2012). In the ‘phyl.pca’ function, we set the method for obtaining the correlation structure as “lambda” and the mode as the correlation matrix.

To assess the phylogenetic signal in the backbone characteristics, Pagel's λ (Freckleton et al., 2002; Pagel, 1999) was calculated. The ‘pgls’ function from the ‘caper’ package was used to calculate Pagel's λ via the maximum likelihood (ML) approach (Orme et al., 2023). The analysis was conducted using the logarithmically transformed average values of the vertebral column characteristics for each species. We utilized Local Moran's index (*I*_*i*_) to estimate local indicators of phylogenetic association (LIPA). The computation of *I*_*i*_ was performed using the ‘lipaMoran’ function, and the results were visualized with the ‘dotplot.phylo4d’ function from the ‘phylosignal’ package (Keck et al., 2016). The significance level was set at *p* ≤ 0.05. It is important to exercise caution when interpreting the results of our phylogenetic analysis, as the number of species studied may be insufficient to obtain reliable estimates of the phylogenetic signal and its significance.

## 4 RESULTS

### 4.1 Formulae optimization

We found that the final values of the K_R_ and K_S_ coefficients for the LB and AR aROM calculation in the RfL division in carnivorans do not differ from those in artiodactyls (Table 1). However, precise SB aROM calculation requires a higher value of the *K*_S_ coefficient in carnivorans compared to artiodactyls (0.27 vs. 0.18). This indicates that in the RfL division, artiodactyls retain relatively greater facet overlap in extremely flexed and extremely extended spine positions than carnivorans. Note that this coefficient value is even higher in humans (*K*_R_ = 0.87 and *K*_S_ = 0.29 in humans vs. *K*_R_ = 1 and *K*_S_ = 0.27 in carnivorans).

### 4.2 Characteristics of the vertebral column

The number of presacral vertebrae in carnivorans (Table 2) is, on average, by one greater than in artiodactyls (26–28 vs. 25–27; mean 26.9 vs. 26.1) and by more than three lower than in perissodactyls (29–32; mean 30.3). In 48 of 56 studied specimens, the vertebral formula consists of 20 thoracolumbar vertebrae (Table S1). We found 19 thoracolumbar vertebrae in 6 specimens (12 T + 7 L in *Viverra zibetha* and two of three studied *Acinonyx jubatus*; 14 T + 5 L in *Ailuropoda melanoleuca* and one of two studied *Hyaena hyaena*; 15 T + 4 L in one of two studied *Crocuta crocuta*). We noted 21 vertebrae (14 T + 7 L) in *C. lupus familiaris* and one of two studied *Nyctereutes procyonoides*.

**FIGURE 2:**
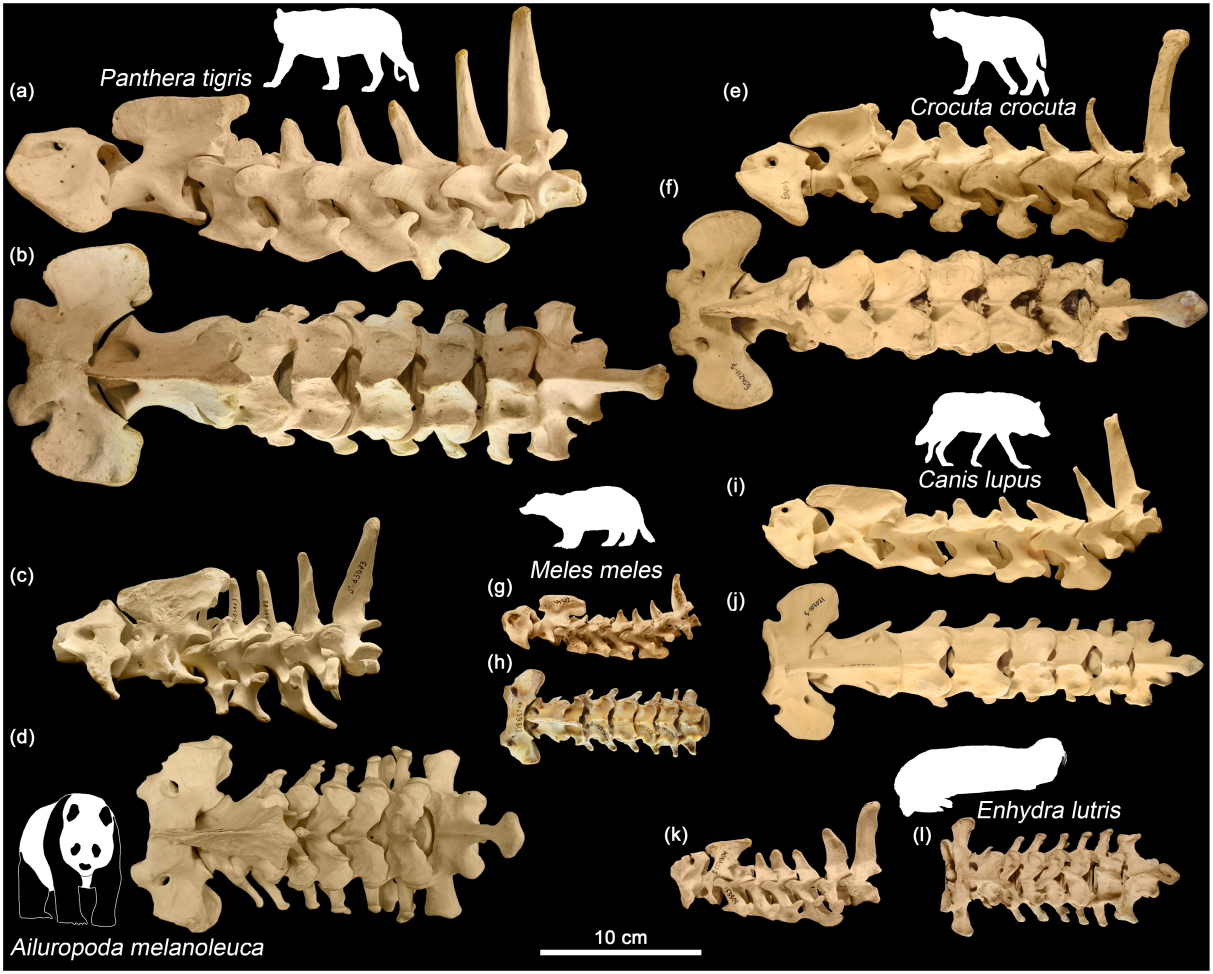
Cervical region (C1‐T1) in various representatives of carnivorans in left lateral (a, c, e, g, i, k) and dorsal (b, d, f, h, j, l) view. (a, b) *Panthera tigris* (ZMMU S‐135467); (c, d) *Ailuropoda melanoleuca* (ZMMU S‐65673); (e, f) *Crocuta crocuta* (ZMMU S‐112459); (g, h) *Meles meles* (ZMMU S‐139312); (i, j) *Canis lupus* (ZMMU S‐107021); (k, l) *Enhydra lutris* (ZMMU S‐153904).

The cervical region in all representatives consists of 7 vertebrae (Figure 2). The transition from the radial type of facets, which is typical for joints starting from C2–C3, to the tangential type of facets in most representatives takes place on the second thoracic vertebra (T2). A more anterior transition from the Rf to the Tf type taking place on T1 was noted only for the sea otter (*Enhydra lutris*) and the Asian black bear (*Ursus thibetanus*). The Rf type in the T1‐T2 joint is also typical for equids and tapirs, and the Tf type in this joint is typical for rhinoceroses and majority of even‐toed ungulates.

**FIGURE 3:**
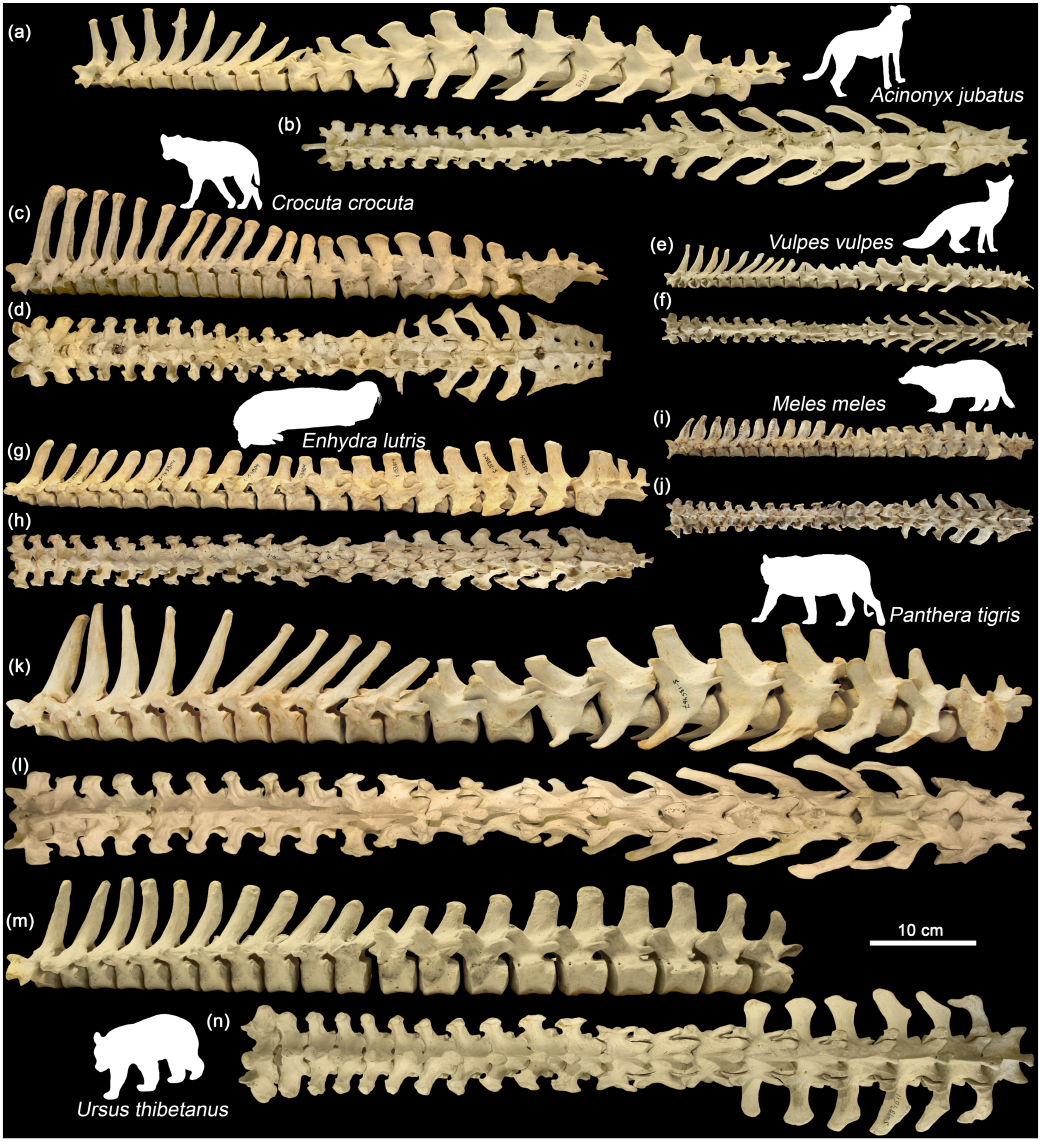
Thoracolumbar part of the backbone (T1‐S) in various representatives of carnivorans in left lateral (a, c, e, g, i, k, m) and dorsal (b, d, f, h, j, l, n) view. (a, b) *Acinonyx jubatus* (ZMMU S‐171619); (c, d) *Crocuta crocuta* (ZMMU S‐112459); (e, f) *Vulpes vulpes* (ZMMU S‐113114); (g, h) *Enhydra lutris* (ZMMU S‐153904); (i, j) *Meles meles* (ZMMU S‐139312); (k, l) *Panthera tigris* (ZMMU S‐135467); (m, n) *Ursus thibetanus* (ZMMU S‐184611).

The thoracic region in carnivorans consists of 12–15 vertebrae, and the lumbar region of 4–7 vertebrae (Table S1; Figure 3). The Kruskal–Wallis *H* test indicated statistically significant differences between carnivoran taxa in the number of thoracic (*χ* ^2^ = 39.741, *p* < 0.001) and lumbar vertebrae (*χ* ^2^ = 46.784, *p* < 0.001). The group with the least numerous thoracic vertebrae and the most numerous lumbar vertebrae includes Felidae, Viverridae and Canidae (***T***: mean from 12.88 to 13.15; ***L***: mean from 6.5 to 7). The group with the most numerous thoracic vertebrae and the least numerous lumbar vertebrae includes Mustelidae and Hyaenidae (***T***: mean 14.23 and 14.75; ***L***: mean 5.77 and 4.75).

Most studied carnivorans are characterized by 3 sacral vertebrae (69.6%). However, post hoc multiple comparisons indicated that Ursidae have a significantly larger number of sacral vertebrae (mean = 5.38; Figure 3).

The transition from Tf to RfL divisions always occurs anterior to the boundary between the thoracic and lumbar regions (Figure 1). The most anterior transition was noted in T9 in two of the three studied cheetahs and *Viverra zibetha*, whereas the most posterior transition was noted in T13 in one of the two studied *Hyaena hyaena* and *Crocuta crocuta* (see Table S1).

The number of Tf joints on average is less than the number of RfL joints (8.80 vs. 10.16; Table 2). In artiodactyls, there is on average one more Tf than RfL joints (10.1 vs. 8.94, respectively); in perissodactyls, the number of Tf joints is notably higher than RfL (17.59 vs. 4.93).

The Kruskal–Wallis *H* test indicated statistically significant differences among various groups of carnivorans in the number of both Tf (*χ* ^2^ = 31.232, *p* < 0.001) and RfL joints (*χ* ^2^ = 35.554, *p* < 0.001). The group with the least numerous Tf and the most numerous RfL joints includes Viverridae, Canidae, and Felidae (***Tf*:** mean 8, 8.15, and 8.31; ***RfL*:** mean 10.5, 11, and 10.56, respectively). The group with the most numerous Tf and the least numerous RfL joints includes Ursidae and Hyaenidae (***Tf*:** mean 9.5 and 10.5; ***RfL*:** mean 9.5 and 8).

In carnivorans, the relative length of the cervical region (C/(T + L + S) ratio) differs by three times (Table 2). In Hyaenidae, the relative neck length (min = 42.6%, mean = 47%, max = 52.8%) is notably longer than in other families. This ratio is relatively large in Canidae (mean = 38.4%). Representatives of Mustelidae, Ursidae and Felidae (mean from 26.1% to 27.5%) have very short necks (Figure 2), similar to the shortest cervical regions in artiodactyls and perissodactyls.

The relative length of the lumbar region in carnivorans, on average, slightly exceeds this ratio in artiodactyls (mean 40.9% vs. 35.3%) and is almost twice as long as that of perissodactyls (mean = 22.1%). In a few studied cheetah specimens, the relative length of the lumbar region exceeds 50% of trunk length. In contrast, in Ursidae, Mustelidae and Hyaenidae, the thoracic region is notably longer than lumbar region (mean from 63.0% to 71.8%; Figure 3).

The relative length of the RfL division in carnivorans, on average, exceeds the length of Tf division (55.4% vs. 41.0%). In artiodactyls and especially perissodactyls, the RfL division length is notably shorter (mean = 46.5% and 20.8%, respectively). Within the carnivoran families, it is notably longer in Canidae, Felidae, and Viverridae (mean 57.8%, 59.7% and 62.1%, respectively), than in Hyaenidae, Ursidae, and Mustelidae (mean 40.7%, 50.5% and 54.2%, respectively; Tables 3 and 4).

**TABLE 3:** aROMs in Rf, Tf, and RfL divisions of the vertebral column and relative length of lumbar region and RfL division in studied Feliformia.

Taxa	Rf cumulative			Tf cumulative			RfL cumulative			L/(T + L) (%)	RfL/(T + L) (%)
	SB (°)	LB (°)	AR (°)	SB (°)	LB (°)	AR (°)	SB (°)	LB (°)	AR (°)		
Felidae	**117.3**	**157.4**	**51.1**	**56.2**	**100**	**97.5**	**95.1**	**88.9**	**21.8**	**47.4**	**59.7**
*Acinonyx jubatus*	113.4	172.1	48.3	51.4	93.1	89.8	107.7	99.8	26.5	52.1	62.4
*Acinonyx jubatus*	92.8	160.4	46.3	45.9	96.0	92.6	101.3	89.7	18.4	48.3	67
*Acinonyx jubatus*	N/A	N/A	N/A	N/A	N/A	N/A	106.2	76.1	19.0	50.3	65.4
*Caracal caracal*	138.4	154.6	49.5	57.5	94.4	91.1	108.3	87.4	10.9	48.7	64.1
*Felis catus*	96.9	132.0	47.3	89.9	113.6	109.4	97.6	110.8	28.1	48.4	58.6
*Felis catus*	127.2	145.0	46.0	63.5	126.2	122.5	108.8	102.3	21.4	49.8	59.6
*Leptailurus serval*	124.9	162.6	50.0	62.8	113.3	109.5	94.2	102.9	25.5	47.6	61.4
*Leptailurus serval*	133.7	167.1	52.3	88.7	103.4	99.3	95.6	99.4	15.8	46.1	60.8
*Lynx lynx*	114.8	151.0	39.0	63.6	114.0	111.0	87.6	85.8	26.3	49.9	62.7
*Panthera leo*	101.8	145.2	55.3	50.8	99.5	95.8	84.5	84.0	26.7	43.6	52.8
*Panthera onca*	141.2	158.1	65.3	53.4	98.5	95.0	93.5	77.4	14.2	44.1	53.8
*Panthera pardus*	110.0	138.1	50.1	59.0	101.4	97.7	89.9	93.2	33.3	46.0	55.6
*Panthera tigris*	114.2	158.1	47.0	50.5	107.1	103.2	90.8	80.0	16.7	44.0	54
*Panthera tigris*	105.0	164.6	55.6	48.7	108.4	104.5	89.1	79.4	11.7	45.7	56
*Panthera tigris*	129.1	165.9	51.9	48.4	83.2	80.1	108.4	94.0	23.9	44.5	60.9
*Panthera uncia*	105.4	147.9	53.6	58.8	101.2	97.7	74.5	95.6	37.3	49.2	59.9
Hyaenidae	**126.2**	**176.4**	**58.2**	**49.2**	**99.7**	**96.5**	**80.6**	**85**	**30.1**	**28.2**	**40.7**
*Crocuta crocuta*	123.7	166.0	61.7	46.8	112.6	108.8	81.7	84.6	21.4	29.6	44
*Crocuta crocuta*	129.2	173.6	55.8	N/A	N/A	N/A	67.6	78.9	29.1	25.2	35.9
*Hyaena hyaena*	127.0	188.3	59.6	55.0	104.0	100.6	75.1	69.9	23.1	29.3	39.3
*Hyaena hyaena*	124.9	177.6	55.7	45.9	82.5	80.0	98.1	106.7	46.8	28.9	43.6
Viverridae	**101.8**	**164.7**	**51.1**	**59.7**	**103.6**	**100.1**	**123.1**	**100.7**	**33.8**	**42.8**	**62.1**
*Arctictis binturong*	78.4	142.9	38.1	72.5	120.6	116.4	120.7	91.7	24.8	38.4	61.6
*Viverra zibetha*	125.4	186.6	64.2	46.8	86.6	83.9	125.6	109.7	42.8	47.3	62.5

*Note*: The mean values in families are highlighted in bold.

**TABLE 4:** aROMs in Rf, Tf, and RfL divisions of the vertebral column and relative length of lumbar region and RfL division in studied Caniformia.

Taxa	Rf cumulative			Tf cumulative			RfL cumulative			L/(T + L) (%)	RfL/(T + L) (%)
	SB (°)	LB (°)	AR (°)	SB (°)	LB (°)	AR (°)	SB (°)	LB (°)	AR (°)		
Canidae	**130.5**	**178.5**	**45.5**	**54.1**	**91.7**	**88.6**	**110.3**	**95.4**	**30.8**	**43.1**	**57.8**
*Canis lupus*	129.8	173.7	48.9	50.7	90.2	87.1	106.8	93.3	36.9	43.6	58.7
*Canis lupus*	141.2	187.6	40.6	49.7	86.6	83.4	124.0	82.5	25.7	42.8	56.1
*Canis lupus*	127.5	176.3	55.1	51.7	90.9	88.0	119.5	85.7	28.7	43.1	58.3
*Canis lupus*	112.8	172.0	40.9	64.1	86.7	83.8	113.1	96.1	39.7	43.2	58.5
*Canis lupus familiaris*	126.6	163.3	48.5	53.6	90.7	87.9	116.7	110.8	44.7	41.7	60.6
*Chrysocyon brachyurus*	93.3	167.1	41.9	52.0	89.1	86.4	84.0	119.4	53.9	43.2	57.9
*Chrysocyon brachyurus*	119.5	175.3	49.5	N/A	N/A	N/A	90.4	107.9	34.0	42.1	56.1
*Cuon alpinus*	125.0	177.6	40.3	34.6	81.1	78.4	88.1	87.5	17.1	43.7	58.7
*Cuon alpinus*	127.8	173.4	43.4	37.3	81.6	78.7	117.8	88.0	19.8	44.0	58.7
*Lycaon pictus*	147.4	197.0	38.7	49.7	91.5	88.5	98.8	96.6	33.2	43.1	57.9
*Nyctereutes procyonoides*	166.3	190.7	52.7	83.3	111.4	107.6	140.3	104.3	31.5	41.5	56
*Nyctereutes procyonoides*	158.4	186.1	52.4	68.7	101.4	97.9	136.0	83.7	11.4	43.9	53.7
*Vulpes vulpes*	121.6	180.1	38.0	54.0	98.7	95.4	98.0	84.8	24.2	43.9	60
Ursidae	**101.8**	**146.2**	**50.2**	**65.3**	**106.8**	**103.2**	**86.9**	**93.7**	**34.2**	**37**	**50.5**
*Ailuropoda melanoleuca*	71.8	105.3	42.6	68.0	98.3	94.6	80.8	73.9	22.0	33.0	44.9
*Tremarctos ornatus*	123.5	182.9	65.2	63.5	113.7	110.1	112.4	95.1	37.6	42.0	52.5
*Tremarctos ornatus*	101.9	143.9	49.7	N/A	N/A	N/A	82.8	107.3	34.5	34.6	47.8
*Tremarctos ornatus*	N/A	N/A	N/A	N/A	N/A	N/A	91.4	97.3	32.9	41.3	52.7
*Ursus arctos*	108.3	149.2	46.5	65.5	98.6	95.0	85.5	110.1	42.3	36.9	56.9
*Ursus maritimus*	93.1	145.3	51.3	60.1	95.8	92.3	84.2	96.8	40.7	34.4	49.1
*Ursus maritimus*	120.1	158.1	55.1	69.6	124.8	120.9	65.4	91.8	40.5	36.0	45.9
*Ursus thibetanus*	93.9	138.7	41.1	65.1	109.9	106.3	92.7	77.3	23.4	38.1	54.1
Mustelidae	**105.8**	**138.5**	**45.7**	**66.8**	**102.3**	**98.7**	**106.5**	**88.1**	**24.7**	**33.5**	**50.7**
*Gulo gulo*	107.0	142.8	43.4	67.9	98.7	95.3	106.6	88.3	27.0	30.3	50.9
*Gulo gulo*	89.5	127.7	42.3	66.3	102.1	98.8	109.2	85.9	29.5	30.4	50.6
*Enhydra lutris*	82.6	111.1	43.2	83.1	127.4	122.7	101.5	79.5	19.6	35.6	45.9
*Martes flavigula*	137.5	164.3	47.3	69.3	100.1	96.7	102.1	89.1	29.4	40.8	55.8
*Meles meles*	112.2	146.7	52.3	47.6	83.0	80.0	113.2	97.7	17.9	30.3	50.3

*Note*: The mean values in families are highlighted in bold.

#### 4.2.1 Fusions in the vertebral column

In our previous studies on ungulates (Belyaev et al., 2021b, 2023), we observed a number of cases of fusion between vertebrae with complete loss of mobility and zygapophysial facet losses. This was noted in 3 of 54 artiodactyls and 7 of 30 in perissodactyls. In this study, we found two carnivoran specimens with fusion between vertebrae and none with zygapophysial facets losses. The fist fusion was noted in the C2‐C3 joint in the giant panda (Figure 2c,d), and the second was found between the last lumbar vertebrae (L5) and the sacrum in the spotted hyena (ZIN 11470; Figure S5). It is interesting to note that in *C. crocuta*, there is a compensatory increase in mobility in the other lumbar joints. So, the average SB aROM in the lumbar joints of ZIN 11470 is ~2° higher than in other studied specimens, and as the result, the cumulative SB aROM in the lumbar region is almost identical (47.1° vs. 49.2°; Table S3).

### 4.3 Rf division

**FIGURE 4:**
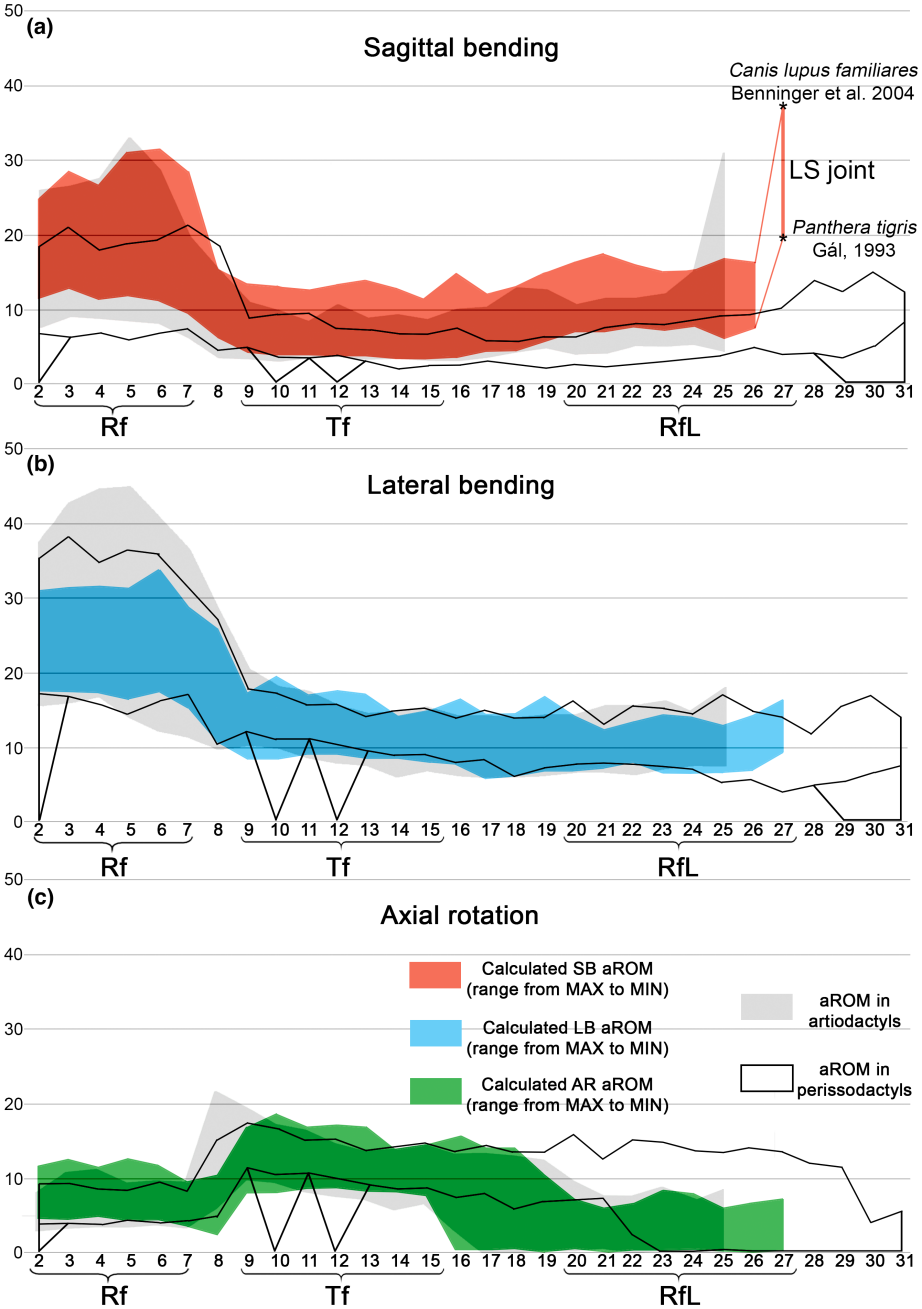
aROM variability (range from maximum to minimum) in the presacral intervertebral joints in carnivorans. (a) SB aROM; (b) LB aROM; (c) AR aROM. Abscissa axis is graduated by joint numbers. For comparison, aROMs previously reported for artiodactyls (Belyaev et al., 2022) are shown as the grey zone and reported for perissodactyls (Belyaev et al., 2023) are shown as the transparent zone.

Joint‐to‐joint aROM values are presented in Table S3. The K‐S test showed that the mobility in the joints of the cervical region in our sample did not have a normal distribution (***SB*:** *n* = 318, *p* = 0.007; ***LB*:** *n* = 317, *p* = 0.024). aROM values in the joints of the cervical region in carnivorans were unevenly distributed (Figure 4). The Kruskal–Wallis *H* test (***SB*:** *χ* ^2^ = 122.274, *p* < 0.001; ***LB*:** *χ* ^2^ = 73.913, *p* < 0.001) indicated that the differences in mean aROM values in particular cervical joints were statistically significant. Post hoc multiple comparisons indicated three or four homogenous subsets of joints in mean SB aROMs. The T1‐T2 joint (mean = 10.2°) is significantly less mobile compared to the intracervical Rf joints (Figure 4). Among most posterior vertebrae, the neck‐thorax joint (C7‐T1) is, on average, notably less mobile than other joints (mean = 14.8°). The C3‐C4, C5‐C6, and C6‐C7 joints are most mobile (mean = 19.2°, 19.1° and 20.3°, respectively), superior to the C4‐C5 and C2‐C3 (mean = 18.1° and 16.2°, respectively). In LB aROM, post hoc multiple comparisons distinguished two groups among the joints of the cervical region: C7‐T2 joints (mean = 21.1° and 19.5°, respectively) are significantly less mobile than more anterior joints C2‐C7 (mean from 23.6° to 25.3°).

#### 4.3.1 Sagittal bending aROM

**FIGURE 5:**
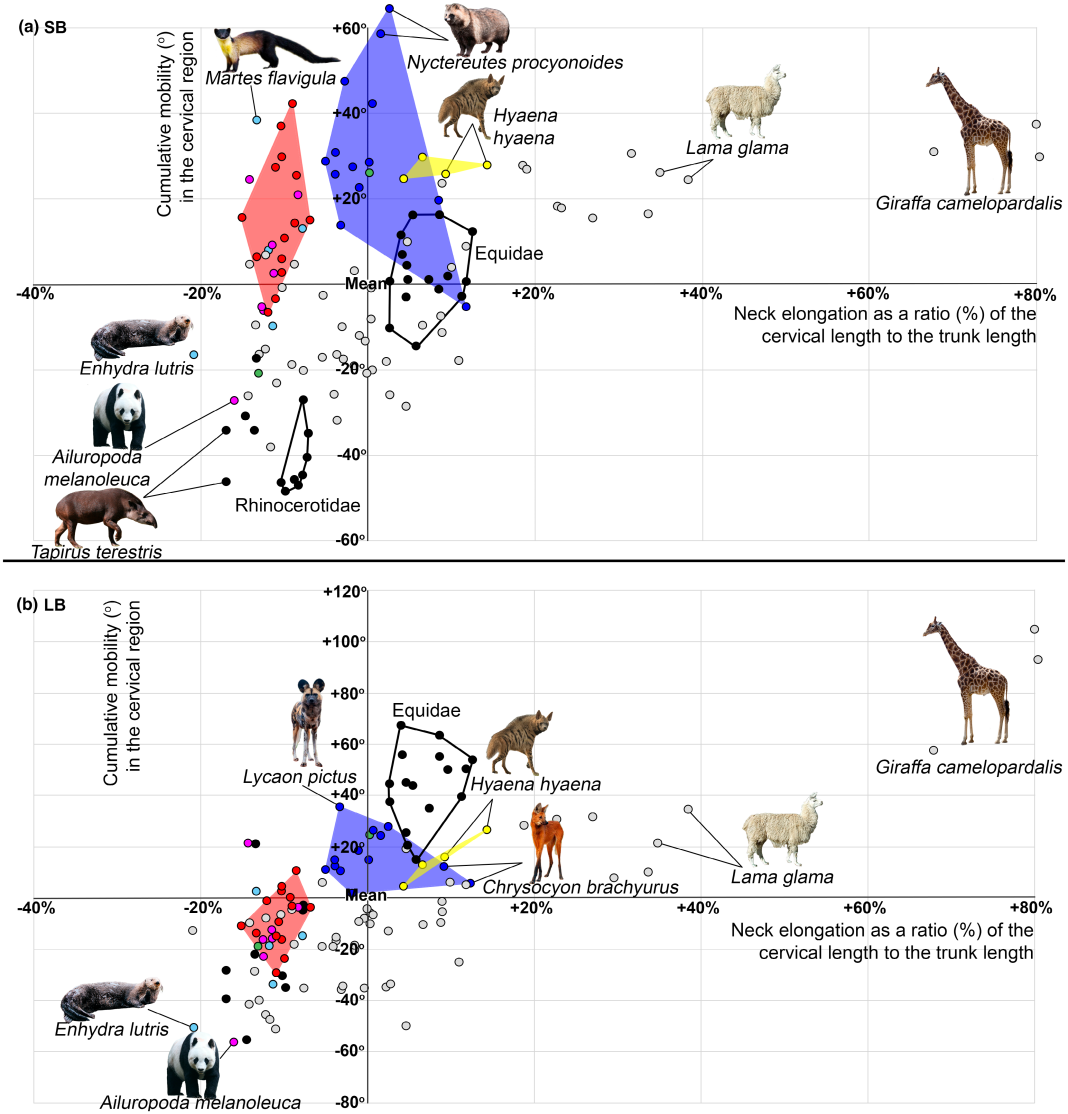
Mobility in the cervical region in carnivorans. (a) SB aROM; (b) LB aROM. Abscissa axis represents the neck elongation as a percentage (%) of the cervical length to the trunk length (T + L + S). Ordinate axis represents the cumulative mobility (°) in the cervical intervertebral joints. Mean values for all studied carnivorans, odd‐toed, and even‐toed ungulate species are taken for the point of axes intersection (neck elongation mean = 37.9%; cumulative SB aROM mean = 99.8°; cumulative LB aROM mean = 162°). The circle colors indicate the taxonomic group: Felidae (red), Canidae (blue), Viverridae (green), Hyaenidae (yellow), large Mustelidae (pale blue), Ursidae (purple). The previously reported data for artiodactyls (Belyaev et al., 2021b) indicate by grey circles, for perissodactyls (Belyaev et al., 2023) by black circles. The list of the studied ungulates is given in Table S1.

The cumulative SB aROM in the Rf division varies by ~95° across carnivorans (Tables 3, 4 and S4.1). ANOVA showed that cumulative SB aROM between carnivorans, odd‐toed and even‐toed ungulates differed statistically significantly (*F* = 31.784, *p* < 0.001). The Rf division in carnivorans has, on average, 25° more SB aROM than in artiodactyls and 35° more than in perissodactyls (Figures 4a and 5a). ANOVA indicated statistically significant differences between various taxonomic groups of carnivorans, odd‐toed and even‐toed ungulates taken together (*F* = 17.356, *p* < 0.001). Post hoc multiple comparisons indicated that Hyaenidae (mean = 126.2°) and Canidae (mean = 130.5°) have highly mobile necks similar to those of Camelidae and Giraffidae (mean 122.1° and 128.3°, respectively). Note that Camelidae have six Rf joints (C2‐T1) while most of carnivorans and giraffes have seven (C2‐T2). Cumulative SB aROMs in other Carnivora families are lower (mean from 101.8° to 116.6°), similar to those of Tragulidae and Tayassuidae (mean = 101.1° and 105°; Figure 5a). However, all carnivorans are significantly superior in cumulative SB aROM to the lowest values observed in even‐toed (pigs, hippos, and bovins; mean 74.3°–80.3°) and odd‐toed ungulates (rhinos and tapirs; mean = 57.4° and 66.6°; Figure 5a).

#### 4.3.2 Lateral bending aROM

The range of cumulative LB aROM in the Rf division varies by more than 90° across carnivorans (Tables 3, 4 and S4.1). ANOVA indicated statistically significant differences in LB aROM in carnivorans, odd‐toed and even‐toed ungulates (*F* = 7.519, *p* = 0.001). The Rf division in carnivorans had, on average, the same degree of LB aROM as artiodactyls (mean = 162° and 153.4°, respectively) and more than 20° less mobility than in perissodactyls (Figure 5b). ANOVA indicated statistically significant differences between taxonomic groups of carnivorans, odd‐toed and even‐toed ungulates taken together (*F* = 24.414, *p* < 0.001). Post hoc multiple comparisons indicated that LB aROM in Hyaenidae (mean = 176.4°) and Canidae (mean = 178.5°) is relatively high, being significantly lower only than in the most mobile necks of ungulates found in Camelidae, Equidae, and Giraffidae (mean 191.9°, 205.4° and 246.5°, respectively; Figure 5b). The cumulative LB aROM in Mustelidae (mean = 138.5°) is relatively low, similar to the short‐necked ungulates like Tapiridae and Rhinocerotidae (mean = 136.7° and 143.7°, respectively). The cumulative LB aROM in Ursidae, Felidae, and Viverridae (mean 153°, 155.8° and 164.7°, respectively) is similar to the Cervidae and large antelopes (mean = 156.2° and 158.5°, respectively).

In carnivorans both the cumulative SB and LB aROM in the Rf division of the vertebral column have a very high positive correlation with elongation of the cervical region relative to the thoraco‐lumbar‐sacral region as a whole (*r* = 0.409 and 0.687, *p* = 0.006 and <0.001). In artiodactyls (*r* = 0.762 and 0.883, *p* < 0.001) and perissodactyls (*r* = 0.856 and 0.876, *p* < 0.001), these correlations are even higher.

#### 4.3.3 Axial rotation aROM

The variability of the cumulative AR aROM across carnivorans is slightly over 25° (38–65.3°; Tables 3, 4 and S4.1). ANOVA showed that cumulative SB aROM in carnivorans, odd‐toed and even‐toed ungulates differed statistically significantly (*F* = 49.657, *p* < 0.001). The Rf division in carnivorans has, on average, 8° more AR aROM than in perissodactyls and 13° more than in artiodactyls. ANOVA indicated a statistically significant difference in cumulative aROMs in various taxonomic groups of carnivorans, odd‐toed and even‐toed ungulates taken together (*F* = 11.988, *p* < 0.001). Overall, AR aROM in Rf joints of carnivorans is less restricted than in ungulates. The highest mobility is a characteristic of Hyaenidae (mean = 58.2°), which markedly exceeds the maximum among ungulates (mean = 43.8° in Equidae).

### 4.4 Tf division

The Tf division is the only part of the vertebral column where AR aROM exceeds SB aROM – by at least 1.1 times, and up to 2.3 times in Hyaenidae However, even there AR aROM does not exceed LB aROM in any case. The cumulative LB and AR aROM in the Tf division are almost identical, with a slight prevalence of LB (~3%).

#### 4.4.1 Sagittal bending aROM

The cumulative SB aROM in the Tf division varies by more than 50° across carnivorans (Tables 3, 4 and S4.2). ANOVA showed that the mean SB aROM in the joints of the Tf division in carnivorans (mean = 6.9°) is slightly, but significantly (*F* = 24. 098, *p* < 0.001), greater than in odd‐toed (mean = 5°) and even‐toed ungulates (mean = 5.6°). ANOVA indicated statistically significant differences between taxonomic groups of carnivorans, odd‐toed and even‐toed ungulates taken together in SB aROM in the Tf division (*F* = 4.645, *p* < 0.001). Mean SB aROM in the Tf division in Canidae and Ursidae (mean = 6.6° and 6.9°, respectively) is similar to the highest in ungulates, while in Felidae, large Mustelidae, and Viverridae (mean = 7.2°, 7.2° and 7.4°, respectively) are the highest among all families.

On average, the cumulative SB aROM in the Tf division in carnivorans is almost the same as in even‐toed ungulates (58.8° vs. 56.5°). The minimum and maximum values depend largely on the number of intervertebral joints. Thus, lower cumulative values in Hyaenidae and Canidae (mean = 49.2° and 54.1°, respectively) are similar to Camelidae, Hippopotamidae, Cervidae, and small antelopes (mean from 47.9° to 52.5°). Higher cumulative values in Ursidae and large Mustelidae (mean = 65.3° and 66.8°, respectively) are similar to Bovini (mean = 65.5°).

#### 4.4.2 Lateral bending and axial rotation aROMs

The cumulative LB and AR aROMs in the Tf division varies by ~45° across carnivorans (Tables 3, 4 and S4.2). The Kruskal–Wallis *H* test indicated that mean LB and AR aROMs in Tf joints in carnivorans, perissodactyls and artiodactyls did not differ significantly (***LB*:** *χ* ^2^ = 3.442, *p* = 0.179; ***AR*:** *χ* ^2^ = 2.254, *p* = 0.324). Due to the smaller number of Tf joints in carnivorans, the cumulative LB and AR aROMs are 15–20° lower than in the artiodactyls and twofold lower than in perissodactyls (***LB*:** 100° vs. 204°; ***AR*:** 97° vs. 197°).

### 4.5 Lumbar region and RfL division

In all carnivorans RfL division includes not only lumbar region but, in addition, 2–5 preceding joints. The RfL division is the only part of the vertebral column where the cumulative SB aROM often exceeds LB aROM (~70% of our sample). On average, the cumulative SB aROM is noticeably exceeds LB aROM in Canidae, Mustelidae, and Viverridae, while in Felidae, Hyaenidae, and Ursidae, these aROMs are relatively close (Tables 3 and 4). The difference between the mean cumulative SB and LB aROM in different carnivoran families does not exceed 10%.

#### 4.5.1 Sagittal bending aROM

Subsequent analysis did not include mobility in the lumbosacral joint because the formula for the RfL division is not suitable to effectively calculate hypermobility in the LS joint. Student's *t*‐test (paired samples) showed that the mean SB aROM in the lumbar region slightly, yet statistically significantly, exceeds SB aROM in the thoracic RfL joints (*n* = 45, Mean Diff = 0.6°, *t* = 2.714, *p* = 0.009, 95% CI: 0.1°–1.0°). Student's *t*‐test (independent sample) indicated that cumulative SB aROM in the thoracic RfL joints in carnivorans significantly exceeds that of artiodactyls (Mean Diff = 12.9°, *t* = 7.642, *p* < 0.001, 95% CI: 9.6°–16.3°). The cumulative SB aROM in the thoracic RfL joints among carnivorans is lowest in Ursidae and Felidae (mean = 24.2° and 24.7°, respectively) and highest in Viverridae and large Mustelidae (mean = 37.0° and 41.1°, respectively).

The cumulative SB aROM in the lumbar region varies by ~60° across carnivorans, and in the RfL division by ~75° (Table S3, S4.3–S4.4). ANOVA indicated statistically significant differences in the cumulative SB aROMs in the lumbar region (*F* = 92.449, *p* < 0.001) and RfL division (*F* = 165.783, *p* < 0.001) between carnivorans, odd‐toed and even‐toed ungulates. On average, the lumbar region in carnivorans is ~23° more mobile than in artiodactyls and ~38° more mobile than in perissodactyls (mean = 33.2°); carnivoran RfL division is ~35° more mobile than in artiodactyls and ~70° more mobile than in perissodactyls (mean = 28.6°).

**FIGURE 6:**
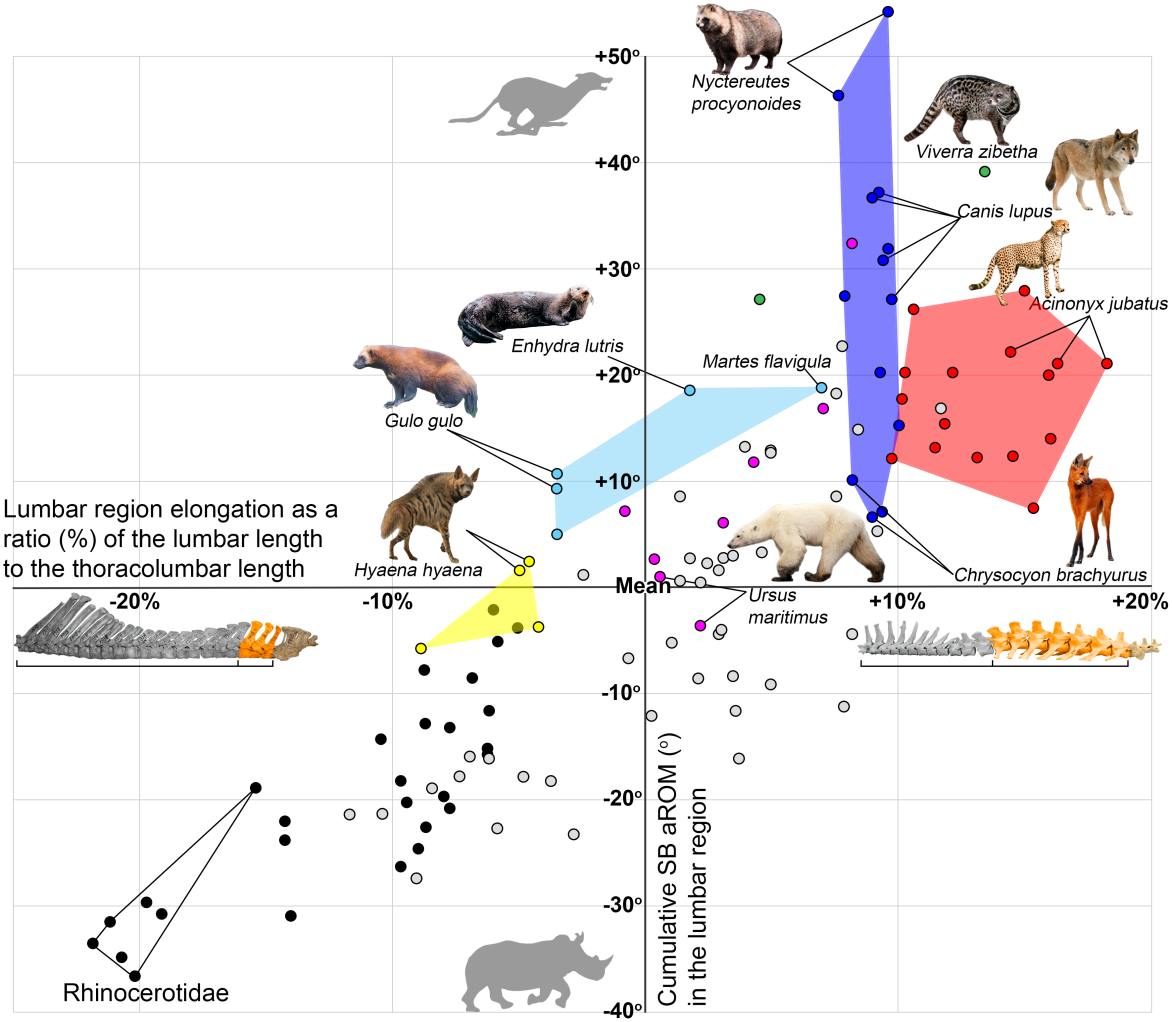
Sagittal mobility in the lumbar region of the backbone in carnivorans. Abscissa axis represents the lumbar region elongation as a percentage (%) of the lumbar length to the thoracolumbar length. Ordinate axis represents the cumulative SB aROM (°). Mean values for all studied odd‐toed and even‐toed ungulate species are taken for the point of axes intersection (lumbar region elongation mean = 34.6%; cumulative SB aROM in lumbar region mean = 53.6°). Circle colors indicate taxonomic groups as in Figure 5.

ANOVA indicated a statistically significant difference in cumulative SB aROM in the lumbar region in various taxonomic groups of carnivorans, odd‐toed and even‐toed ungulates taken together (*F* = 25.021, *p* < 0.001). The lowest cumulative SB in the lumbar region among carnivorans is characteristic of hyenas (mean = 53.1°): it is at the same level as relatively mobile lumbar regions in such artiodactyls as Cervidae and Caprinae (mean = 49.6° and 54.8°, respectively; Figure 6). The cumulative SB aROM in Ursidae and large Mustelidae (mean = 62.7° and 65.4°, respectively) is at the same level as the most mobile lumbar regions in artiodactyls (small antelopes mean = 61.1°). The cumulative SB aROM in Felidae (mean = 70.8°) is similar to the maximum among ungulates in Tragulidae (mean = 70.7°). The cumulative SB aROM in Canidae and Viverridae (mean = 80.4° and 86.2°, respectively) is even higher (Figure 6).

In carnivorans the cumulative SB aROM in the lumbar region of the vertebral column has a very high positive correlation with elongation of the lumbar region relative to the thoracolumbar region as a whole (*r* = 0.465, *p* = 0.001). This correlation is even higher in artiodactyls (*r* = 0.778, *p* < 0.001) and perissodactyls (*r* = 0.845, *p* < 0.001).

#### 4.5.2 Lateral and axial rotation aROMs

The range of cumulative LB aROM in the RfL division varies by ~50° across carnivorans (Table S4.4); AR aROM in the RfL division varies by ~40° (Table S4.4). Student's *t*‐test (independent sample) showed that both mean and cumulative LB and AR in the lumbar region (and RfL division) in carnivorans and artiodactyls do not differ significantly. The most restricted AR in the lumbar region in carnivorans is characteristic of large Mustelidae and Felidae (cumulative AR mean = 14.2° and 14.8°, respectively), and the least restricted AR is found in Ursidae (mean = 25.2°).

### 4.6 Phylogenetic analysis

**FIGURE 7:**
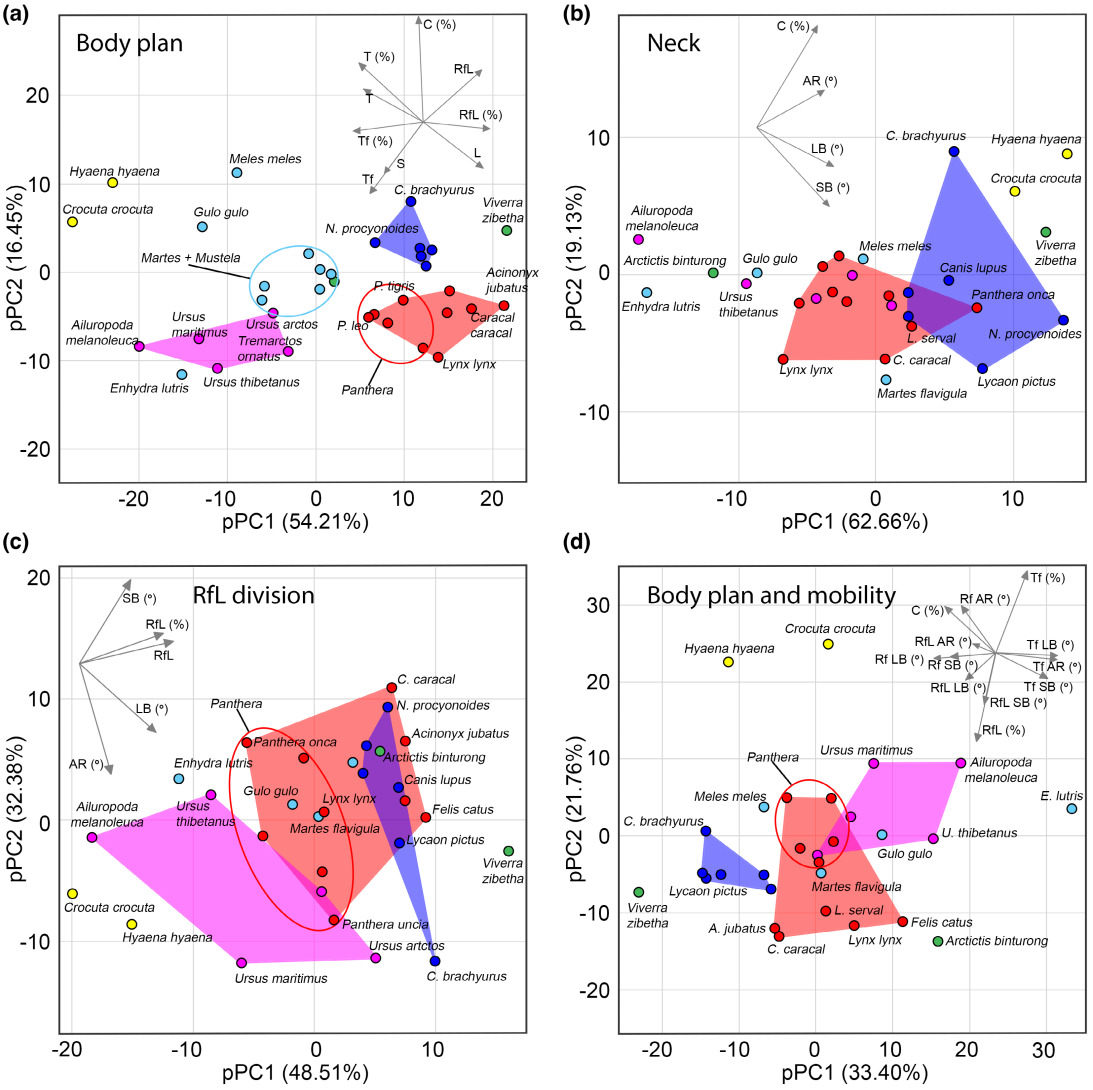
Phylogenetic PCA biplots. Numerical characteristics and ratios of the vertebral column (a); cervical region elongation and mobility (b); RfL division (~posterodorsal module) elongation and mobility (c); ratios and mobility of the vertebral column divisions (d). Circle colors indicate taxonomic groups as in Figure 5.

We observed a strong phylogenetic signal (λ ≥ 0.9) in the majority of numerical characteristics and length ratios of the vertebral column in carnivorans (Table S6). However, among all characteristics of intervertebral mobility, only the cumulative LB aROM in the Rf division exhibited a high and significant phylogenetic signal (Table S6). The LIPA analysis revealed significant Local Moran's index (*I*_*i*_) values for LB aROM in the Rf division in Canidae, as well as for the relative length of the cervical region in both Hyaenidae and Canidae. Meanwhile, the phylogenetic signal in all three directions of cumulative intervertebral mobility in the lumbar region and RfL division of the backbone did not significantly differ from zero (Table S6). The pPCA was performed four times using different variables. A detailed description of the pPCA analysis performed is presented in SuppInfo S7; the pPCA biplots are presented in Figure 7.

## 5 DISCUSSION

### 5.1 Backbone regionalization

Narita and Kuratani (2005) showed that the vertebral formula in stem mammals probably consists of 7 cervical and 19 thoracolumbar vertebrae. Carnivora deviates from this plesiomorphic state toward 20 thoracolumbar vertebrae. This state is very consistent and probably acquired from common ancestor of extant carnivorans (Narita & Kuratani, 2005). This is in agreement with our observations that 48 of 56 studied specimens are characterized by 20 thoracolumbar vertebrae (Table S1). The vertebral formula is very stable in studied Felidae and Canidae and consists of 13 T + 7 L (with four exceptions mentioned in Section 4.2). In Ursidae, it deviates towards 14 T + 6 L, in mustelids towards 14‐15 T + 5‐6 L, and in hyenas towards 14‐15 T + 4‐5 L (Table S1).

Despite the stability of the vertebral formula, the boundaries and proportions of the functional modules of the vertebral column vary noticeably (Figure 7a). The cervical region in carnivorans is functionally elongated due to the T1‐T2 joint, which is characterized by Rf type zygapophyses, like in the neck. A similar specialization of the T1‐T2 joint is a characteristic of equids and the giraffe (Belyaev et al., 2023). In both cases, such zygapophysial connections correspond to a significant increase of SB aROM in the T1‐T2 joint (Gunji & Endo, 2016; Townsend et al., 1983). However, in carnivorans, SB aROM in the T1‐T2 joint is on average only ~2.5–3° more mobile than thoracic Tf joints beginning with T2‐T3. In LB aROM, this difference is more notable (~7°). Overall, in the Rf division in carnivorans, the cumulative LB aROM exceeds SB aROM (by 1.1–1.8 times) and SB aROM exceeds AR aROM (by 1.7–3.8 times).

**FIGURE 8:**
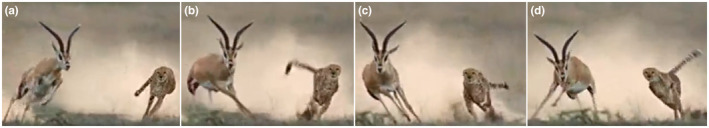
The Grant's gazelle (*Nanger granti*) changes its running direction in an attempt to trick the cheetah (*Acinonyx jubatus*) chasing it (from https://www.youtube.com/watch?v=qukcc8wCxJo).

The boundary between the anterodorsal and posterodorsal modules of the backbone (Martín‐Serra et al., 2021) is almost exactly coincident with the Tf‐to‐RfL zygapophysis transition. The anterodorsal module (~Tf division) is believed to be related to the motion constraints of the thorax related to breathing (Filler, 1986; Martín‐Serra et al., 2021). If so, the anterodorsal module should provide only static support during locomotion. However, in our previous study, we showed the kinematic significance of the Tf division (Belyaev et al., 2021b). The fact that AR has considerably expanded amplitudes in anterodorsal module of backbone manifests that this direction of mobility is of primary importance in this part of the body. AR aROM in the Tf division exceeds SB aROM in every studied joint of every studied specimen (by 1.1–2.3 times) and approaches LB aROM. Expanded AR in this part of the body has a variable use in mammalian locomotion, including the peculiar archaic walking of monotremes (Gambaryan & Kuznetsov, 2013), self‐grooming for thermoregulation (Jones et al., 2020), and maneuvering. AR‐capable Tf joints allow tilting of the parasagittal plane of the forelimbs to one side or the other relative to that of the hind limbs and vice versa. For instance, independent rolling of the fore and hind halves of the body to the left or right side allows smart cornering in hares (Kuznetsov et al., 2017). One can hypothesize that the primary and most general input of the increased AR mobility to fitness in cursorial mammals is that it ensures agile maneuvering (Figure 8). Indeed, the increased AR aROM was found in Tf division both in artiodactyls and perissodactyls (Townsend et al., 1983; Wilke et al., 2011; Wilke, Kettler, & Claes, 1997). Perissodactyls exceed artiodactyls in this respect due to the markedly increased number of Tf joints. Surprisingly, carnivorans on average show lower cumulative AR aROM in Tf division than in ungulates.

The posterodorsal module (~ RfL division) of the backbone is related to the ability to flex and extend in the sagittal plane, which is actively involved during bounding gaits. This module includes not only lumbar but also some posterior thoracic joints. As shown by Schilling and Hackert (2006), in various small mammals the SB‐capable module of the backbone is not exactly associated with either the lumbar region or RfL division. In some species (*Dasyuroides byrnei*, *Galea musteloides*), the anteriormost joint with a large SB uROM during galloping is in the anterior part of the lumbar region. However, in other species (*Ochotona rufescens*, *Tupaia glis*, *Monodelphis domestica*), not only the lumbar joints but also some of the thoracic RfL joints are actively involved during gallop. Published data on intervertebral mobility in domestic pig (Busscher et al., 2010; Wilke et al., 2011) shows that RfL‐thoracic joint is characterized by increased SB amplitudes compared to those in the middle third (Tf joints) of the thoracic region. Studied carnivorans are characterized by 2–5 RfL‐thoracic joints. The most numerous RfL joints are characteristic of Viverridae and Canidae (mean 10.5 and 11, respectively), the least numerous RfL joints are characteristic of Ursidae and Hyaenidae (mean 9.5 and 8). The RfL division is the only part of the backbone where SB aROM values can exceed the two other directions of mobility. In most specimens, SB is either equal to LB or slightly exceeds it, up to 1.6 times. AR aROM in RfL joints is markedly restricted and significantly inferior to both LB and SB aROM.

The weakly integrated transitional (diaphragmatic) vertebra with Tf prezygapophyses and RfL postzygapophyses forms the transition between the antero‐ and posterodorsal modules. SB aROM in the first RfL joint (the joint between the diaphragmatic vertebra and first posterodorsal vertebra) is on average ~ 1° lower than in the rest of the thoracic RfL joints and lumbar RfL joints. This difference is not expressed in studied Viverridae, Hyaenidae, and Ursidae, and is well expressed in Felidae, large Mustelidae, and Canidae (mean diff between first RfL joint and lumbar joints is ~ − 1°, −1.5°, and − 2.6°, respectively).

In carnivorans, the RfL division is on average longer than the Tf division (~55% of thoracolumbar length vs. ~ 41%, respectively). The relative length of the RfL division and lumbar region (~55% and ~41%) in carnivorans, on average, slightly exceeds these ratios in artiodactyls (~47% and ~35%, respectively) and significantly exceeds them in perissodactyls (~21% and ~22%). The relative length of the lumbar region in cheetahs (~50%) is almost twofold longer than in equids (~26%; Belyaev et al., 2023).

The change in orientation (anticlination point) of the vertebral spinous processes from a backward to forward inclination in carnivorans may or may not coincide with the change in the zygapophysial facet type (Figure 3). In most species, these transitions are in good agreement with each other. In Ursidae, the spinous anticlination point is not expressed at all (see Figure 3m and https://doi.org/10.6084/m9.figshare.22652104). It is interesting to note that in ursids, the transverse processes of the lumbar vertebrae are laterally oriented rather than cranially (craniolaterally) oriented as in other carnivorans (Figure 3). Contrary to the majority of artiodactyls (Belyaev et al., 2021b,c; Gambaryan, 1974), the tips of the spinous processes in carnivorans are relatively narrow and not extended in an anteroposterior direction. The spinous processes of the vertebrae are the base for attachment of the ligaments preventing excessive sagittal flexion. Therefore, in carnivorans, the interspinal ligaments connecting the spinous processes are relatively long and the supraspinal ligament is either poorly developed or absent (Gambaryan, 1974). Longer ligaments are able to stretch more, which allows for increased ventral flexion. If these assumptions are correct, then ventral flexion of the posterodorsal spine in carnivorans should markedly exceed that of ungulates. We will return to this issue at the end of Section 5.4.

#### 5.1.1 Phylogenetic signal

The strong phylogenetic signal observed in numerical characteristics and length proportions of the vertebral column parts (Table S6) may be indicative of neutral evolution (neutral genetic drift) or evolutionary conservatism in these traits (Kamilar & Cooper, 2013). The latter could result from factors such as stabilizing selection, random fluctuations of natural selection, low rates of evolution, or strong physiological/morphological constraints (Kamilar & Cooper, 2013). This shows that the structural basis of the backbone (vertebral formula, joint formula, elongation of regions and divisions) in carnivorans is very stable in large taxonomic groups. The high homogeneity of the structural basis of the vertebral column in extant representatives of different carnivoran families probably indicates that its stabilization in different phylogenetic lineages occurred rather early.

The non‐significant phylogenetic signal in the mobility of the lumbar region and in most characteristics of aROM in general could indicate high rates of evolution in these traits, leading to substantial differences among closely related species (Kamilar & Cooper, 2013). Apparently, these vertebral column characteristics are crucial in the context of ecological diversification among carnivorans. The diversity in their lifestyles has led to divergence in the mobility of vertebral column, rather than the numerical characteristics and length ratios of its parts. This indicates that, within the existing structure, intervertebral mobility in carnivorans is quite flexible.

### 5.2 Mobility of the neck and how carnivorans catch their prey

The two main prey‐catching strategies that carnivorans utilize are the use of their paws or their jaws. Felidae are well known for their curved, sharp, retractable claws. Felids are able to use their paws both to seize their prey and to knock it down during pursuit. On the other hand, large endurance runners such as hyenas and canids are characterized by the use of their jaws as a tool for seizing prey. This leads us to the question of whether the biomechanical properties of the neck are different in these animals.

The pPCA biplots show that Hyaenidae and Canidae are shifted towards a significantly longer and more mobile cervical region (Figure 7b). Hyaenidae are characterized by the longest neck among carnivorans (mean = 47% of T + L + S length), which is almost similar in relative length to the equids (Figure 5). In Canidae, a similar neck length is characteristic of the peculiar long‐legged maned wolf (46% and 50.8%), while in other species, it is somewhat shorter (~33%–40%). Both the cumulative SB and LB aROM in the cervical region in Canidae and Hyaenidae are the highest in carnivorans (Figure 5). This is the third case of an increase in relative neck length accompanied by an increase in SB and LB flexibility, similarly to Giraffidae and Camelidae among artiodactyls (Belyaev et al., 2021b) and Equidae among perissodactyls (Belyaev et al., 2023). The mobility of the neck in Canidae and Hyaenidae is lower only than the most mobile necks of ungulates found in Camelidae, Equidae, and Giraffidae (Belyaev et al., 2021b, 2023; Clayton & Townsend, 1989; Gunji & Endo, 2016; Stolworthy et al., 2015).

In general, carnivorans that catch prey with their jaws are characterized by a markedly longer cervical region (1), an elongated facial part of the skull (2), and increased flexibility of the neck in the sagittal and horizontal planes (3). The relatively long and more agile neck of Canidae and Hyaenidae probably plays an important role for these animals as a manipulator during hunting.

Representatives of Mustelidae, Ursidae, and Felidae (mean from 26.1% to 27.5%) have very short necks (Figures 2, 5 and 2, 5) similar to the relatively shortest cervical regions in artiodactyls and perissodactyls (Belyaev et al., 2021b, 2023). Felidae's neck is on average 10–14° less mobile than Canidae and Hyaenidae in SB aROM and 22–24° less mobile in LB aROM. It is interesting to note that the short neck of felids is on average (**SB:** ~116°; **LB:** ~155°) markedly more mobile than the short necks of artiodactyls (**SB:** ~75–80°; **LB:** ~120° in pigs and hippos) and perissodactyls (**SB:** ~55–65°; **LB:** ~135–145° in tapirs and rhinos) and very similar in this regard to the small and short‐necked Tragulidae (**SB:** ~100°; **LB:** ~150°).

Representatives of studied Ursidae, Viverridae, and large Mustelidae are characterized by relatively short necks with average flexibility in SB and LB and are mostly similar to Felidae. An interesting case is the sea otter (*Enhydra lutris*). This semi‐aquatic mammal is characterized by the shortest (~18% of T + L + S length) and one of the least mobile (**SB:** ~80°; **LB:** ~110°) necks among all ungulates and carnivorans that we have studied (Figure 5). Shortening of the relative neck length is a characteristic of aquatic mammals such as whales and sirens (Sokolov, 1986). The opposite case is characteristic of otariids, which are characterized by relatively long neck, which they use to initiate manoeuvres during underwater locomotion (Ray, 1963).

The phylogenetic signal in our sample of carnivorans is strong (λ = 1) for both relative neck length and LB aROM in the cervical region. Considering that Hyaenidae and Canidae were hotspots of positive phylogenetic autocorrelation (Figure S8) in the relative neck length and LB aROM in Rf division, this suggests that neck elongation and increased mobility likely evolved early in the history of these clades. Such adaptations may be linked to their prey‐catching strategy.

### 5.3 Mobility of the vertebral column and how carnivorans run

#### 5.3.1 Felids

The vertebral column in Felidae is unique (Figure 7a). It is characterized by the longest lumbar region among all carnivorans and ungulates we have studied (Figure 6). The shortest lumbar regions in felids (~44% of T + L length) are at the same level of elongation as the longest lumbar regions in other carnivorans, and the longest ones are the longest in general (~50%). The sagittal flexibility of the lumbar region in felids is very high, at the level of the most flexible lumbar regions in even‐toed ungulates (Figure 6). Big cats of the genus *Panthera* differ markedly from other representatives of Felidae and are characterized by a shorter and less mobile lumbar region and posterodorsal module of the backbone (Figure 7a,c,d). Mobility in the lumbosacral joint in big cats (~19° in tiger and jaguar; Gál, 1993) is also lower than in the domestic cat (~26°; Jones et al., 2020). Both the lumbar region's elongation and high flexibility of the vertebral column are ways to increase the path of the animal's center of gravity when the lumbar region extends from the maximally flexed position to the straight position, for instance, before a jump.

The most interesting case of running among the felids is the cheetah. Cheetahs are well‐known as the fastest land animals (Wilson & Mittermeier, 2009). Their rotary gallop involves two types of suspension – extended and gathered (in the first one, the limbs are extended maximally away from the middle of the body, the back is straightened, and in the second one, the limbs are gathered under the body, the back is bent; Hildebrand, 1977; Makarov & Panyutina, 2022), and it is also characterized by a relatively small vertical fluctuation of the center of mass (Kamimura et al., 2022). Finally, cheetahs are one of the prime examples of dorsomobility (Gambaryan, 1974; Hildebrand, 1959).

Both lengths of the lumbar region and RfL division (~posteriodorsal module) in cheetah are the longest in Carnivora (Figures 6 and 7a, Tables 3 and 4). The elongation of the SB‐capable part of the backbone is accompanied in the cheetah by an increase in the mass of both the spinal erectors (up to 23.8% of mass of fore‐ and hindlimbs vs. 10.2% to 20.8% in other Felidae) and flexors (m. psoas minor + m. iliopsoas is 7.6% vs. 4.7% to 6%, respectively; Gambaryan, 1974).

**FIGURE 9:**
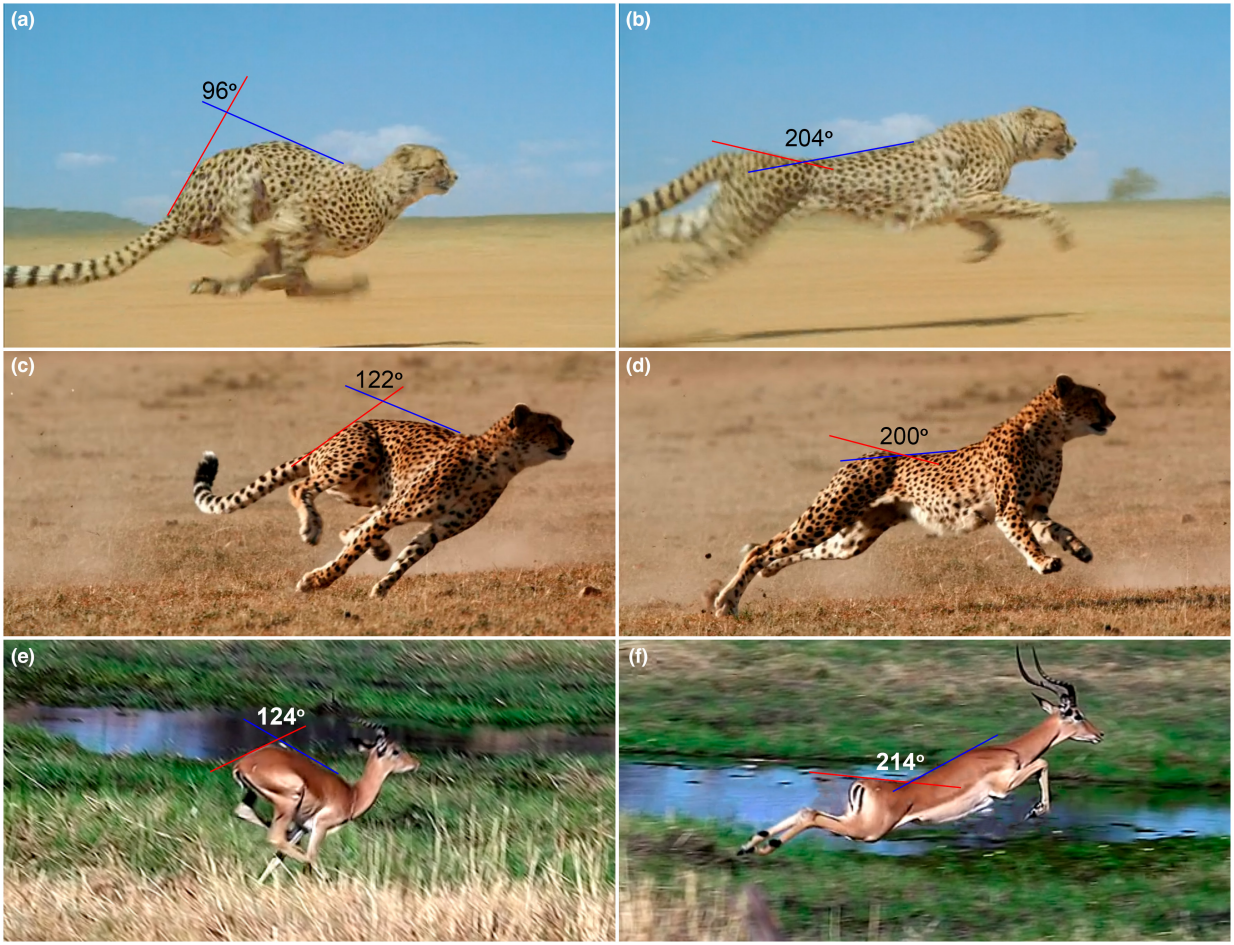
Maximum and minimum sagittal curvature of the lumbosacral part of the backbone during the gallop in (a, b) cheetah running at maximal speed in a straight line (from https://www.gettyimages.de/detail/video/cheetah‐running‐fast‐across‐desert‐unspecified‐stock‐videomaterial/137817399); (c, d) cheetah chasing prey (from BBC The Hunt. Ep.5. “Nowhere to Hide (Plains)”); (e,f) impala jumping (from https://www.gettyimages.de/detail/video/impala‐male‐running‐along‐khwai‐river‐at‐okavango‐stock‐videomaterial/481002647). The difference between the maximum and minimum angles of the curvature represents SB uROM; it equals 108° in (a, b), 78° in (c, d), and 90° in (e, f).

The cumulative SB aROMs in the lumbar region (~75°) and RfL division (~105°) of the backbone of cheetahs are among the highest in Felidae (Figure 6). A potential 20–30° of SB mobility in the lumbosacral joint provides cheetahs with ~100° of mobility in the lumbar spine and ~ 130° in the RfL division. Running in a straight line at top speed, the cheetah is able to use almost full SB aROM of the posterior module of the backbone (~110°, Figure 9a,b). However, during an actual cheetah hunting, the maximum values of backbone flexibility that we were able to fix are ~80° (Figure 9c,d). This can either be an artifact due to an insufficient number of analyzed video, or it can have a real behavioral basis. So, only moderate speeds are involved during the most of cheetah's hunts (Wilson et al., 2013). Probably, this is due to maneuvering while running; the highest speed does not allow the cheetah to change running direction sufficiently.

#### 5.3.2 Canids

The pPCA biplots show that the canid body plan and vertebral mobility are relatively homogenous (Figure 7). Our data indicate that the cumulative SB aROMs in the lumbar region (mean = 84.8°) and RfL division (mean = 116°) in *C. lupus* are very high (Figure 6). These values are higher than in the fox and African wild dog (on average by ~12–15° in the lumbar region and by 17–18° in the RfL division). An additional 28–37° of mobility at the lumbosacral joint (Benninger et al., 2004; Pylypchuk, 1975) provides the wolf and domestic dog with an impressive ~115–120° of mobility in the lumbar region and ~ 145–150° in the RfL division.

**FIGURE 10:**
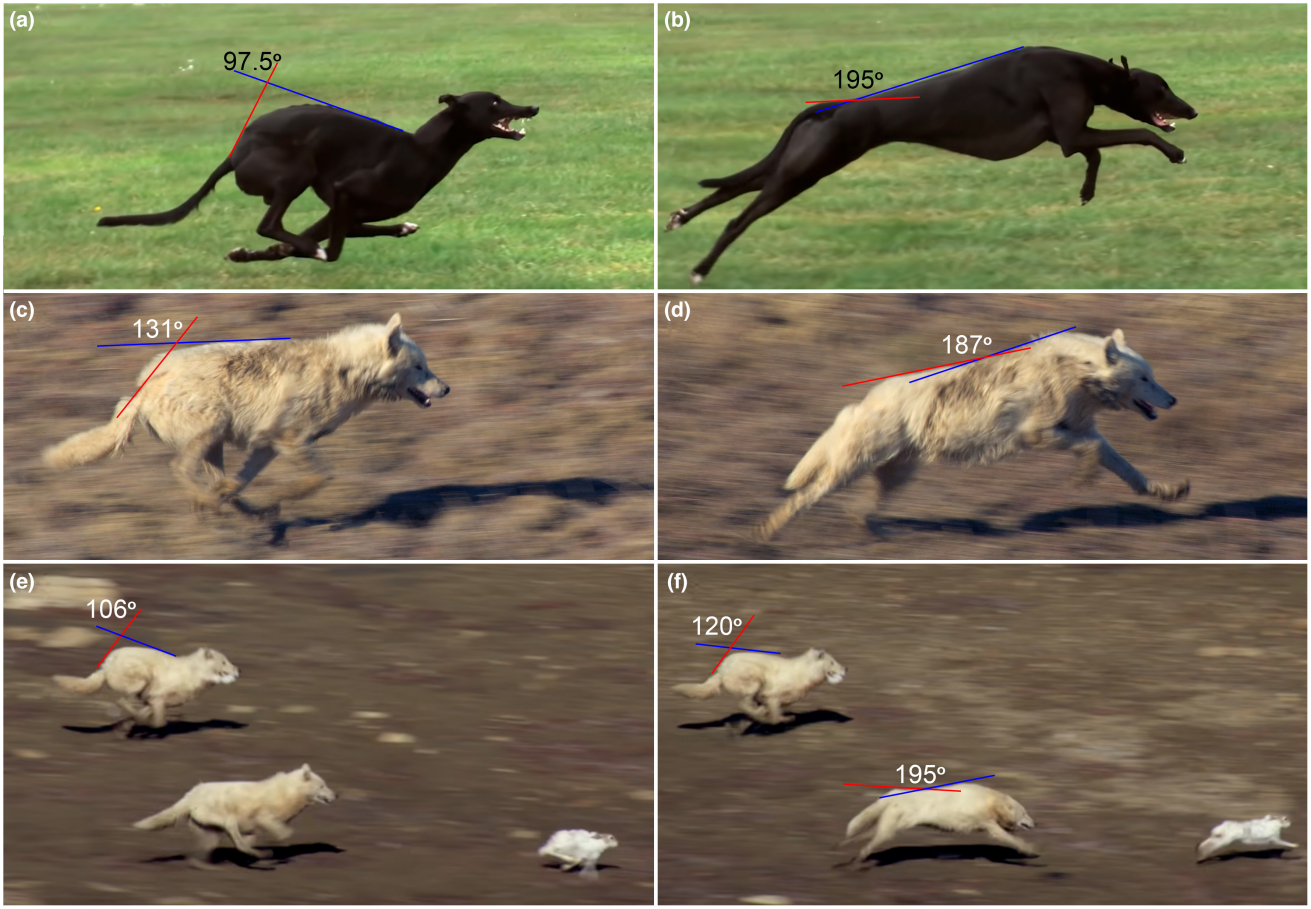
Maximum and minimum sagittal curvature of the lumbosacral part of the backbone during the gallop in Canidae (a, b) greyhound running at maximal speed in a straight line (*Canis lupus familiaris*; from https://www.youtube.com/watch?v=sf9prgDBeAU); (c, d) wolf (*Canis lupus*) chasing hare (from BBC The Hunt. Ep.2. “In the Grip of Seasons Arctic”); (e, f) wolves catching hare (same as c, d). The difference between the maximum and minimum angles of the curvature represents SB uROM; it equals 97.5° in (a, b), 56° in (c, d), and 89° in (e, f).

During fast straight‐line galloping the greyhound is able to engage ~100° of SB mobility in the posterodorsal module of the backbone (Figure 10a,b). During an actual hunt both the wolf and African wild dog combine a pack‐hunting strategy with an endurance gallop, which is almost dorsostable (~40–60°; Figure 10c,d). However, in key moments of the hunt associated with the direct seizure of the prey and acceleration after maneuvering, canids engage the available SB mobility (~90° Figure 10e,f). Substantial involvement of lumbar flexibility in the wolf gallop corresponds to an increase in spinal erector mass compared to the other canids (Gambaryan, 1974). So, in the wolf their mass is 19% of the total mass of fore‐ and hindlimb muscles versus 17.3% in the African wild dog and 15% in the common raccoon dog.

Surprisingly, the largest SB in the lumbar region (100–110°) and the largest SB in the RfL division (135–140°) of the backbone in our sample are characteristics of the common raccoon dog. This small, short‐legged, and relatively robust canid inhabits forested areas and is characterized by an omnivorous diet and is definitely not advanced in running (Gambaryan, 1974; Sillero‐Zubiri, 2009). We can hypothesize that high dorsomobility is characteristic of many small‐sized carnivorans, which is partially supported by the data on the pine marten we studied (see Section 5.3.5).

The most different in body plan and mobility from other canids is the long‐legged maned wolf (Figure 7). The relative length of the lumbar region in this animal is similar to that in the other canids. However, the mobility of the lumbar region (59.5° and 63.7°) and RfL division (84° and 90.4 °) in *Chrysocyon brachyurus* is noticeably lower than in other Canidae (Figure 6). This is somewhat similar to the adaptation of long‐legged artiodactyls (moose, okapi, giraffe) to a more dorsostable gallop (Belyaev et al., 2021b, 2022), which Gambaryan (1974) named a stilt running form.

#### 5.3.3 Hyaenids and viverrids

**FIGURE 11:**
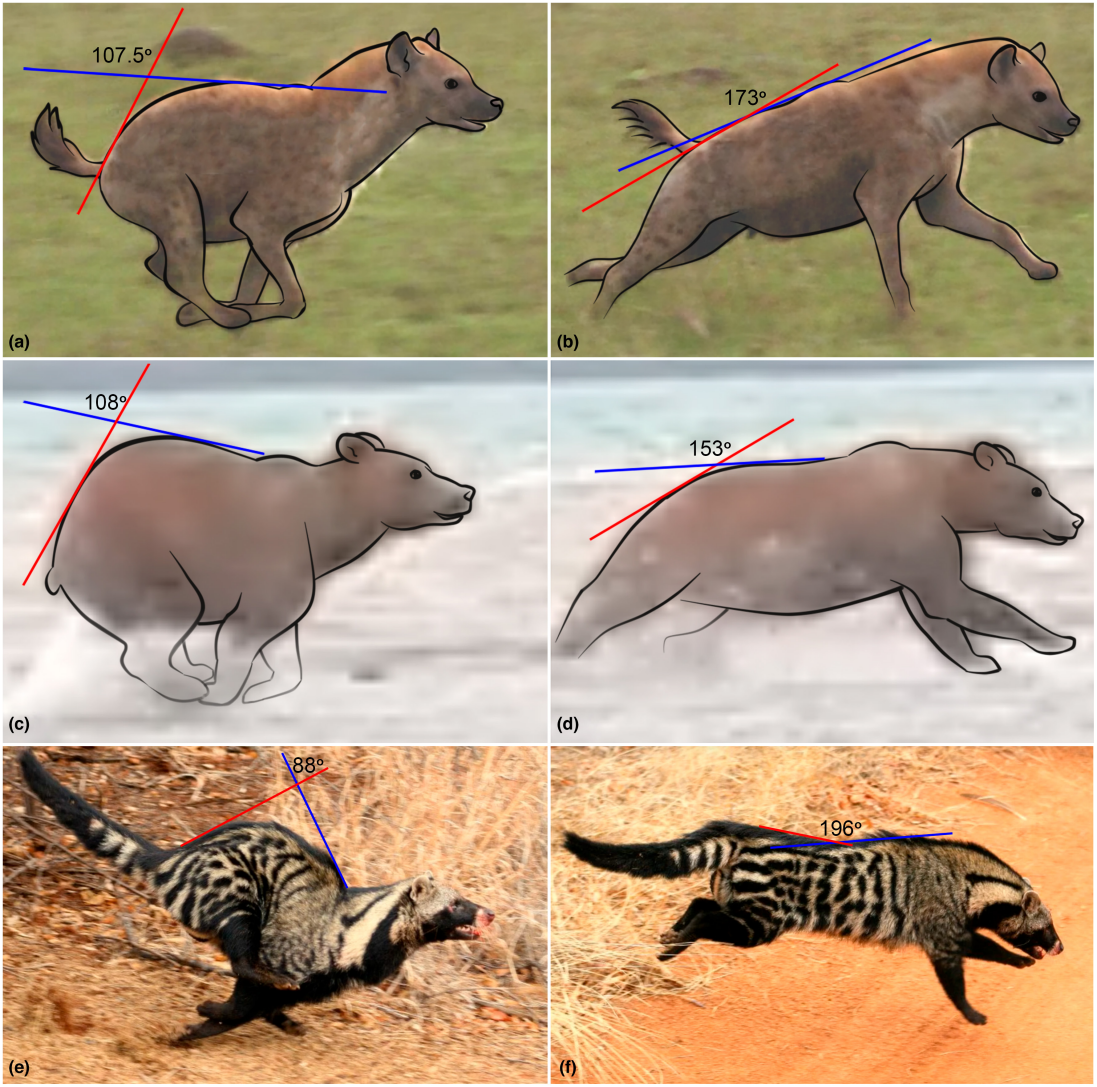
Maximum and minimum sagittal curvature of the lumbosacral part of the backbone during the gallop in (a, b) the spotted hyena (from https://www.gettyimages.de/detail/video/cheetah‐versus‐hyena‐stock‐filmmaterial/603956718); (c, d) the brown bear (from https://www.gettyimages.com/detail/video/alaska‐katmai‐national‐park‐brown‐bear‐running‐stock‐video‐footage/1375‐62); (e, f) the African civet (*Civettictis civetta*; from https://www.youtube.com/watch?v=GvNV5XFlgrg). The difference between the maximum and minimum angles of the curvature represents SB uROM; it equals 65.5° in (a, b), 45° in (c, d), and 108° in (e, f).

The pPCA biplots show that hyenas are characterized by a unique body plan and vertebral mobility for carnivorans; they do not overlap with any of the other studied species (Figure 7). The disproportion in the length of the forelimbs and hindlimbs (Spoor, 1985) makes the hyena's gallop look somewhat peculiar. However, despite this feature, the spotted hyena (*Crocuta crocuta*) can rich a maximum speed of over 60 km/h, and the striped hyena (*Hyaena hyaena*) up to 50 km/h (Kruuk, 1972; Spoor & Belterman, 1986). In general, hyenas are characterized by the development of a more dorsostable gallop than in other carnivorans. This corresponds well to their hunting and chasing strategies. Thus, the spotted hyena, well known as an active predator that hunts as much as lions do (Kruuk, 1972), is able to chase their prey over long distances (up to 24 km; Kruuk, 1972; Mills & Hofer, 1998). Similarly to the hyenas, in ungulates, an adaptation to the endurance gallop in open landscapes (cursorial running form) is accompanied by smaller lumbar and lumbosacral SB aROMs (Belyaev et al., 2021b, 2022, 2023). Similarly to the ungulates (including giraffe and bison, which are also characterized by significant disproportion between fore‐ and hindlimbs lengths), the lumbar region in hyenas becomes relatively shorter and less mobile (Figure 11a,b). Cursorial running mode in hyenas is very effective, so, unlike the other large predators of Africa, spotted hyenas do not preferentially prey on any species. Depending on the parts of its range and the season, *C. crocuta* prefers to hunt a variety of small, medium, and large‐sized ungulates (Hayward, 2006; Holekamp et al., 1997). Evidence of dental microwear suggests that a hypercarnivorous diet was also characteristic of some extinct hyenas (Rivals et al., 2022). The main difference between hyenas and canids, that might have bearing on their endurance chasing strategies, is an additional SB amplitude in canids which possibly helps in the final deadly rush.

In this study, we examined mobility of the backbone only in few individuals of Viverridae. The viverrids we studied are characterized by a notably higher than average SB aROM in the RfL division and lumbar region (Figures 6 and 11e,f). However, the small sample size does not allow us to assess whether high sagittal flexibility is characteristic of all representatives of the group and correspond to their lifestyle.

#### 5.3.4 Ursids

The pPCA biplots show that representatives of the Ursidae are shifted toward a shorter and less SB‐capable posterodorsal module of the backbone (Figure 7c,d). The least SB mobile lumbar region is a characteristic of the largest representative of extant Ursidae – the polar bear (49–54°). Available videos of galloping bears allow us to suggest that the SB aROM is reduced due to restriction of dorsal extension, not ventral flexion (Figure 11c,d). The lumbar region of a bear operates during gallop in a more flexed position than in other carnivorans, and does not straighten even in the extended stage of the locomotor cycle (Figure 11d). Reduced backbone flexibility is accompanied by a decrease of spinal erectors mass in ursids: 10.1% of total mass of fore‐ and hindlimbs muscles in *Ursus arctos* compared with 19% in *Canis lupus* (Gambaryan, 1974).

#### 5.3.5 Mustelids

The pPCA biplots show that typical representatives of Mustelidae (genus *Martes* and *Mustela*) adapted to hunt their prey in burrows and in tree branches are characterized by an almost uniform body plan. This applies both to the small least weasel (*Mustela nivalis*) and the medium‐sized yellow‐throated marten (Figure 7a). However, outside the genera *Martes* and *Mustela*, the body plan of mustelids is extremely flexible and diverse, including relatively robust omnivorous and carnivorous terrestrial animals such as the badger and wolverine, as well as semiaquatic representatives of subfamily Lutrinae (Figure 7).

**FIGURE 12:**
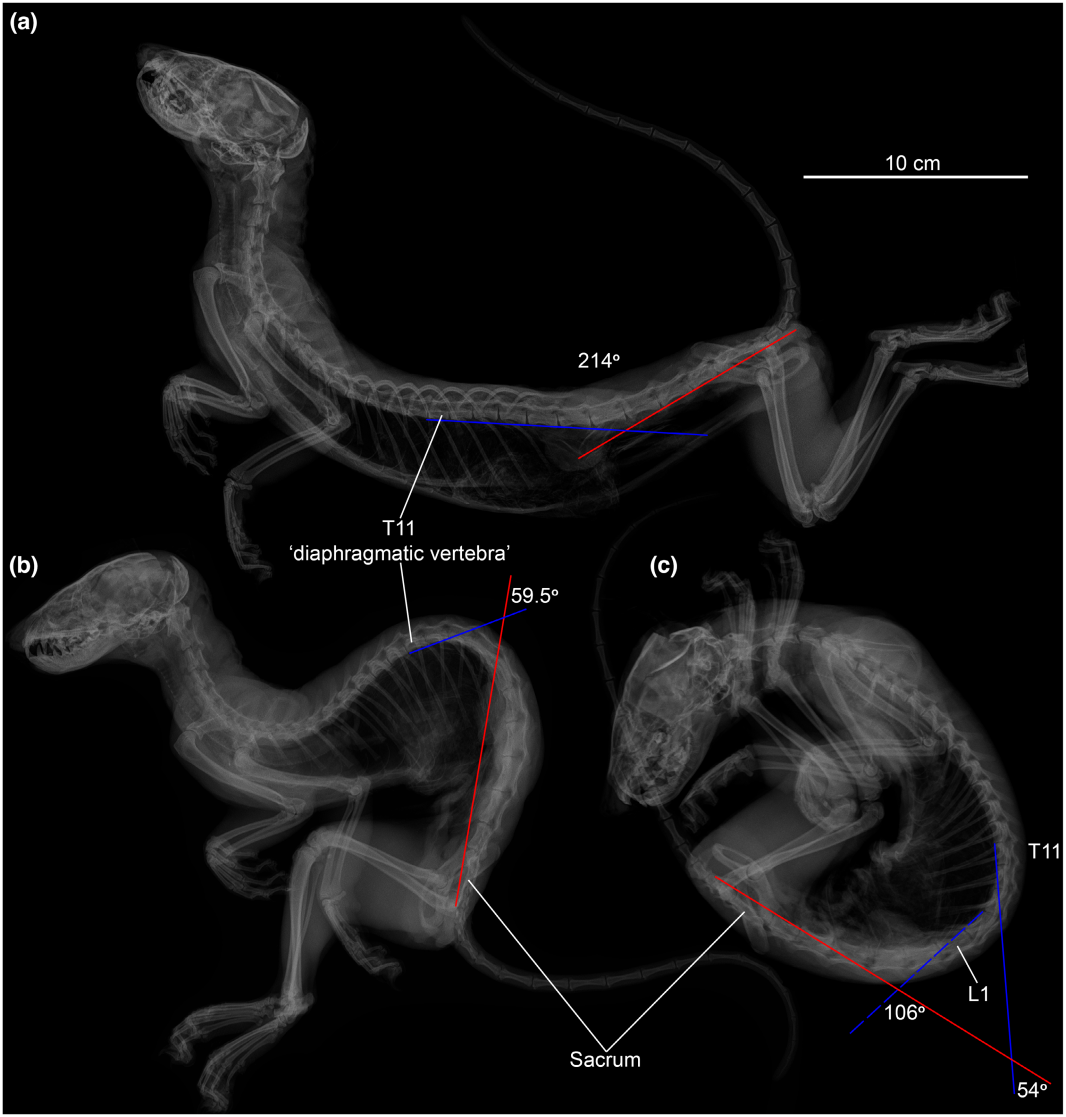
X‐ray images of the *Martes martes* (PAN‐160) in left lateral view: (a) dorsal extension; (b, c) ventral flexion. Lines are drawn along the ventral margin of the certain vertebral body and sacrum. In (c) we measured two angles: between T11 and the sacrum and between the last thoracic vertebra (T14) and the sacrum; the first case corresponds to the cumulative SB aROM in the RfL division and the second to the cumulative SB aROM in the lumbosacral region. The difference between the maximum (a) and minimum (c) angles equals 108° in the lumbosacral region and 160° in the RfL division.

According our X‐Ray data small‐sized mustelids are characterized by an extremely SB‐flexible backbone (Figure 12). The cumulative SB aROM of the lumbosacral region in the pine marten is ~110°, and that of the RfL division is 160°. The increased vertebral excursion is supported by the increased mass of spinal erectors in Mustelidae compared to the other carnivorans: 25% of mass of fore‐ and hindlimbs muscles in *Mustela putorius*, 25.2% in *Martes zibellina*, and 36.2% in *Enhydra lutris* (Gambaryan, 1974). It is important to note that a substantial part of the SB aROM is associated with the thoracic RfL joints (~50° in T11‐T14; Figure 12c). This corresponds well with the finding that, in large Mustelidae, the cumulative SB aROM in the thoracic‐RfL joints is highest in our sample (mean = 41.1°). This indicates that at least in some carnivorans, the posterior thoracic joints are SB‐capable and probably play an important role during locomotion. Moreover, in the pine marten, the first SB‐capable vertebral joint exactly coincides with the fist RfL joint.

The wolverine and badger are characterized by robust build unusual for mustelids. Their thoracic region is significantly longer than the lumbar (70% vs. 30%). The cumulative SB aROM in the lumbar region (without mobility in the LS joint) of the wolverine and badger (~60°) is ~10° lower than in the yellow‐throated marten and sea otter. It can be assumed that this is a result of the characteristic endurance hunting mode of the wolverine and omnivorous digging lifestyle of the badger (Lariviere & Jennings, 2009). Both these adaptations do not require the animals to have a highly flexible backbone. However, the cumulative SB aROM in the RfL division in this species (~110°) exceed these values in the *M. flavigula* and *E. lutris* (~102°). This probably indicates that high SB‐flexibility of the backbone in mustelids is a common feature of the group and remains quite high even in species that have shifted from active hunting toward an omnivorous, less mobile lifestyle.

Special attention should be paid to the sea otter, whose body plan is characterized by the shortest neck among studied carnivorans and the second shortest RfL division. The pPCA biplot shows (Figure 7d) that vertebral mobility in the sea otter is significantly shifted toward enhanced LB and AR aROM in the Tf division. This is probably related to the specificity of movement and maneuvering in the three‐dimensional continuous medium of water.

### 5.4 Are carnivorans actually dorsomobile compared to ungulates?

**FIGURE 13:**
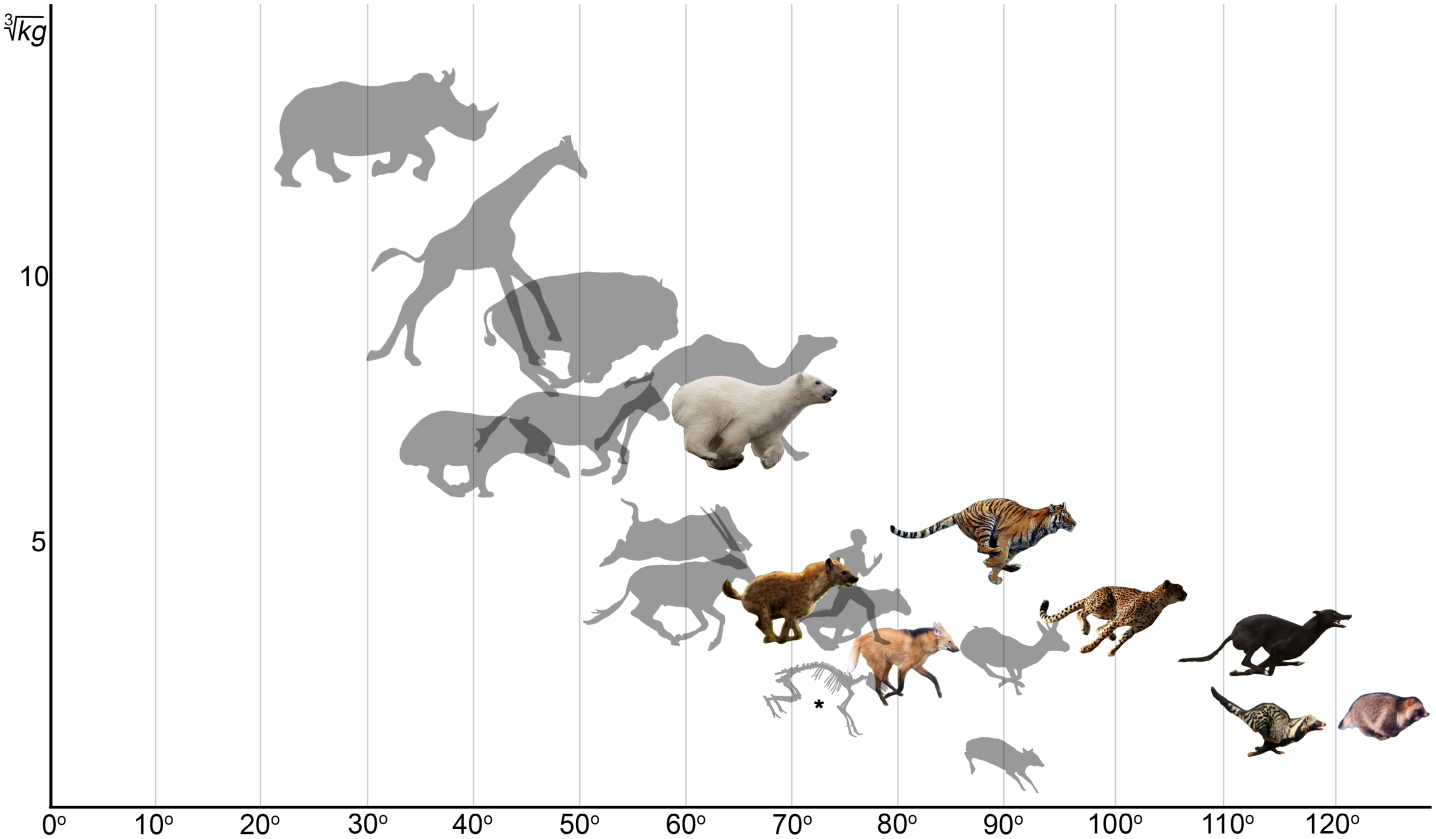
Interrelation between sagittal mobility in the lumbosacral part of the backbone and the cube root of body mass in certain typical representatives of carnivorans, odd‐toed and even‐toed ungulates. Body masses were taken from Wilson and Mittermeier (2009, 2011). *Arenahippus grangeri skeleton modified from https://umorf.ummp.lsa.umich.edu/wp/specimen‐data/?Model_ID=1675.

Gambaryan (1974) suggested the idea of ungulates and carnivorans as “dorsostable runners” and “dorsomobile runners”, respectively. Indeed, as previously shown (Belyaev et al., 2021b, 2022), many small‐ and medium‐sized artiodactyls are characterized by high amplitudes of SB in the lumbar and lumbosacral joints and can be considered as “dorsomobile runners”. Are carnivorans superior to ungulates in vertebral flexibility, and are all of them dorsomobile? In general, carnivorans are indeed more dorsomobile than ungulates. The lumbar region in carnivorans is on average ~23° more mobile than in artiodactyls and is ~38° more mobile than in perissodactyls (RfL division is ~35° and ~70° more mobile, respectively). On the other hand, the cumulative SB in the lumbar region in hyenas is similar to that in Cervidae and Caprinae. In Ursidae and large Mustelidae, it is at the same level as in small antelopes. Finally, in Felidae, it is at the same level as in Tragulidae (Figure 13).

The material studied indicates that fusions between vertebrae and zygapophysial facets losses are markedly less characteristic of carnivorans than ungulates. In our study on perissodactyls, we hypothesized that restricted mobility can be related to vertebral fusion (Belyaev et al., 2023). Probably, the more mobile backbone of carnivorans is less susceptible to pathologies than that of ungulates.

In general, the gallop of carnivorans is characterized by a substantially greater use of SB mobility than the gallop of extant perissodactyls. Thus, carnivorans and perissodactyls can be opposed to each other as “dorsomobile runners” and “dorsostable runners.” In the case of artiodactyls, however, the situation is more complicated. Mobility in the lumbosacral joint is the most notable in this regard. SB aROM in the LS joint differs in artiodactyls almost sevenfold, from 4.5° to 31° (Belyaev et al., 2022). While carnivorans are markedly different, published data show that the lowest known SB aROM in the LS joint is a characteristic of the representatives of genus *Panthera* (~19°; Gál, 1993), and the highest known SB aROM in the LS joint is a characteristic of domestic dog (~37°; Benninger et al., 2004). So, the lowest SB in the LS joint in carnivorans is four times higher than in the most dorsostable artiodactyls, while the highest SB aROM in the LS joint in carnivorans is only 20% greater than the most dorsomobile artiodactyl (Figure 4). Thus, all carnivorans, many small‐ and medium‐sized artiodactyls (Belyaev et al., 2021b, 2022), as well as the Paleogene ancestors of perissodactyls (Belyaev et al., 2023), can be rightfully regarded as “dorsomobile runners,” while all modern‐day perissodactyls and most large artiodactyls can be considered as “dorsostable runners” (Figure 13).

Finally, a comparison of posterodorsal module mobility during galloping in dorsomobile artiodactyls and carnivorans shows that the most dorsomobile ungulates are able to engage even more dorsal extension than carnivorans (Figure 9e,f). However, carnivorans are far superior to ungulates in their ability to employ ventral flexion, as was predicted in Section 5.2 based on their morphology. This may be related to the enlargement of the abdomen in herbivores. Further evidence of this fact is presented in Figure S9 and illustrated by the example of a sheep jumping (Figure S9e,f), the stoat galloping (Figure S9c,d), and the author's dog (Figure S9a,b).

Traditional understanding of dorsomobility‐dorsostability opposition implies SB aROM. However, another component of mobility, the AR aROM, appears to be worthy of mention when opposing ungulates and carnivorans. In the Tf division alone, the average cumulative AR aROM of carnivorans is exceeded by 20% in artiodactyls and more than twice in perissodactyls. Furthermore, the thoracolumbar region as a whole (Tf + RfL divisions with the exception of LS joint) is characterized by the following relations of cumulative SB and AR aROMs: in carnivorans SB is greater than AR by 35°, while in ungulates the balance is reversed with the excess AR of 13° in artiodactyls and 90° in perissodactyls. Overall, ungulates are more dorsostable than carnivorans in regard of thoracolumbar SB aROM, but are more dorsomobile in regard of AR aROM. Therefore, assuming that the backbone AR is a key for maneuvering with parasagittal legs, one can further hypostasize that, in the modern state of predator–prey competition among mammals, carnivorans stake on enhancement of forward acceleration and velocity by means of backbone SB flexibility, while ungulates stake on enhancement of sharp cornering from side to side by means of backbone AR. This hypothesis could be verified in the field with the help of accelerometry and precise GPS tracking of the predator and prey simultaneously.

## 6 CONCLUSION

Despite stability of the number of vertebrae in carnivorans, the boundaries and proportions of the functional modules of the vertebral column vary notably. For instance, the elongation of the lumbar region (relative to the thoracolumbar length) in the cheetah is almost twice as long as in hyaenids. In contrast, the cervical region of hyaenids is almost twofold longer than in felids. These properties in carnivorans depend largely on the mode of running and hunting. We were able to show that carnivorans that seize their prey with their jaws (canids and hyaenids) are characterized by a significantly elongated neck and increased mobility in SB and LB compared to other carnivorans.

The lumbar region in carnivorans is very mobile in the sagittal plane and may be actively involved during galloping. On average, the carnivoran lumbar region is ~23° more mobile than in artiodactyls and ~38° more mobile than in perissodactyls. Despite this, the most dorsomobile artiodactyls are superior to carnivorans in their ability to engage dorsal extension during the gallop. However, carnivorans are far superior to ungulates in their ability to use ventral flexion. Finally, all ungulates, are superior to carnivorans in terms of available axial rotation.

Various carnivorans use an almost dorsostable gallop frequently, even during hunting. In some species (including hyaenids, large ursids, and the peculiar maned wolf), it is associated with reduced mobility. Truly dorsomobile species, represented by large canids and felids, tend to engage the available SB mobility only in the key moments of hunting associated with the direct capture of their prey and acceleration after maneuvering.

## AUTHOR CONTRIBUTIONS

R.B., D.K., and N.P. worked with material, R.B. wrote initial manuscript, R.B., A.B., and A.P. analyzed the data, R.B. and A.B. prepared tables and supplements; R.B., A.B., and A.P. revised the manuscript, and R.B. and P.N. prepared figures. All authors edited the paper, and read and approved the final version of the manuscript.

## CONFLICT OF INTEREST STATEMENT

None.

## Supporting information


**Table S1** Studied material.

**Figure S2.1–S2.3** The scheme of the mechanistic model for calculation formulae of intervertebral aROM based on dimensions of vertebrae (Figure S2.1). Vertebral measurements (Figure S2.2). Examples of different values of the formulae coefficients K_S_ and K_R_ (Figure S2.3).

**Table S3** aROM values in the intervertebral joints of the studied carnivorans.

**Table S4.1‐S4.4** aROMs in Rf, Tf, and RfL division and lumbar region of the vertebral column in carnivorans

**Figure S5** Fusion between L5 and sacrum in *Crocuta crocuta* (ZIN 11470). Dorsal (a) and ventral (b) view.

**Table S6** Pagel’s λ values in certain numerical characteristics, ratios and aROM values of the vertebral column.

**Table S7** Phylogenetic PCA analysis description: variables used; eigenvalues and percentage of variance for the pPCA axes; key variables related to the pPC1 and pPC2 axes.

**Figure S8** Local Moran’s index (I_i_) values for each species for the relative neck length (S8.1) and the cumulative LB aROM in the Rf division (S8.2). Red points indicate significant I_i_ values.

**Figure S9** Maximum and minimum sagittal curvature of the lumbosacral part of the backbone in (a‐b) *Canis lupus familiaris*; (c‐d) the stoat (*Mustela erminea*) chasing hare (from https://www.youtube.com/watch?v=HNbqvqf3‐14); (e‐f) sheep jumping. The difference between the maximum and minimum angles of the curvature represents SB uROM; it equals 110° in (a‐b), 115° in (c‐d), and 93° in (e‐f).

## Data Availability

14 figures with photographs of the vertebral column of the studied carnivorans. All figures: cervical region (C1‐T1) in the left lateral (A) and dorsal (B) views; thoracolumbar and sacral region in the left lateral (C) and dorsal (D) views. Belyaev, Ruslan (2023): Figures of various carnivorans vertebral column. figshare. Figures. https://doi.org/10.6084/m9.figshare.22652104
