# Supplementary material for: Running, jumping, hunting, and scavenging: Functional analysis of vertebral mobility and backbone properties in carnivorans
Source: J Anat. 2023 Oct 14;244(2):205–31. doi: 10.1111/joa.13955 (PMC10780164; doi:10.1111/joa.13955)
Supplement: Supplementary file 2 — Figure S2.1–S2.3 The scheme of the mechanistic model for calculation formulae of intervertebral aROM based on dimensions of vertebrae (Figure S2.1). Vertebral measurements (Figure S2.2). Examples of different values of the formulae coefficients KS and KR (Figure S2.3). [file JOA-244-205-s005.pdf]

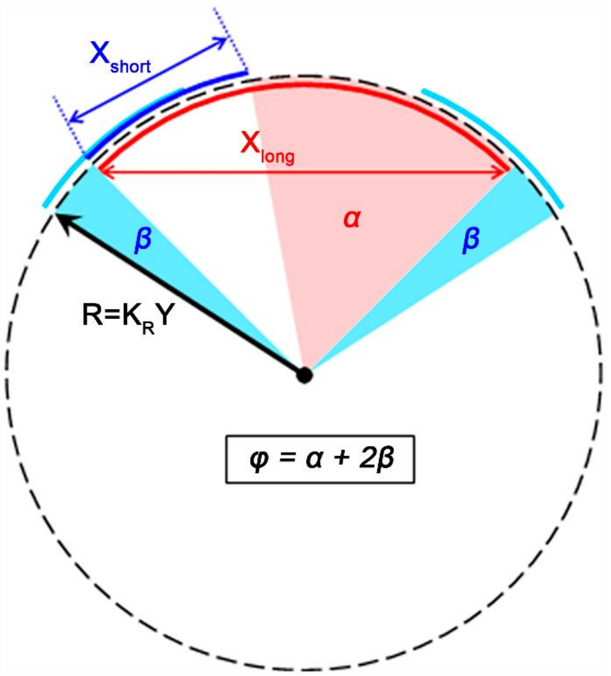

Fig. S2.1 The scheme of the mechanistic model for calculation formulae of intervertebral aROM based on dimensions of vertebrae. In the plane of motion, the zygapophysial facets of the two adjacent vertebrae are treated as arcs of equal radius R (black arrow). One facet is usually smaller (blue arc) than the other (red arc), and their sizes are measured by respective chords X_{short} and X_{long}. The radius R of the facet arcs' curvature is treated as the radius of joint rotation and is derived from one or the other dimension Y of the vertebrae with the multiplication coefficient K_R, which is subject to empirical adjustment. Finally, aROM consists of two terms. The first one represents the available shift of the smaller facet in the limits of the larger one, which is equal to the angular difference *α* between the larger and the smaller facets (pale red sector). The second term represents the available shift of the smaller facet beyond the larger one to every side, which is equal to angular overhanging *β* of the smaller facet in the marginal positions (two pale blue sectors).

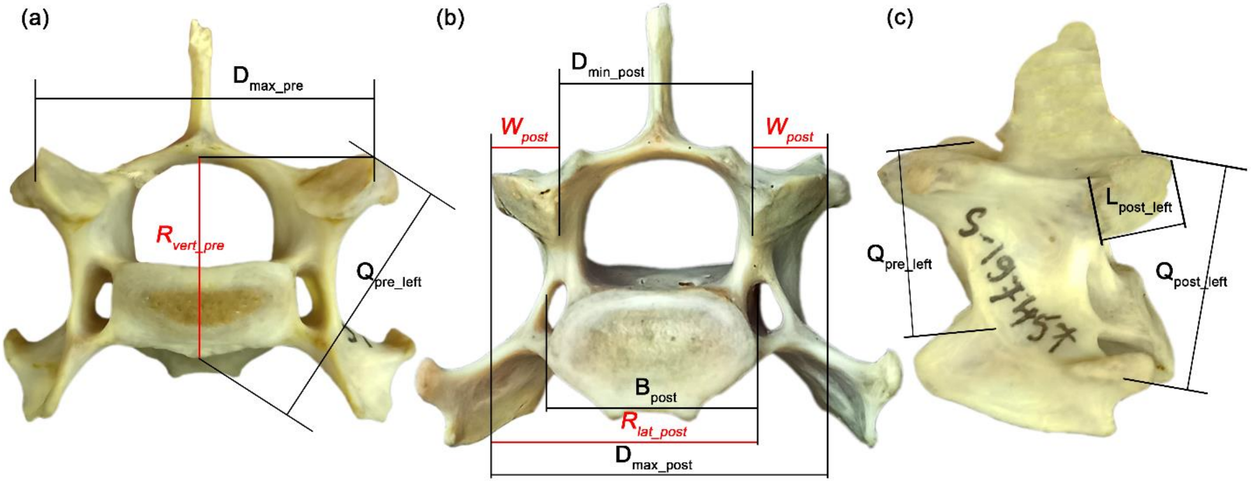

Fig. S2.2 Vertebral measurements. *Lynx lynx* (ZMMU S-197457) vertebra C4 (Rf type) depicted in the anterior (a), posterior (b), and left lateral (c) view. The measurements and the derived parameters (highlighted in red, italics) are shown, which are involved in aROM calculation formulae presented in Table 1.

$W_{pre} = (D_{max\_pre} – D_{min\_pre})/2$ $\qquad$ $W_{post} = (D_{max\_post} – D_{min\_post})/2$

$R_{lat} = B + (D_{max} – B)/2$ $\qquad$ $R_{vert} = \sqrt{(Q^2 - D_{max}{}^2/4)}$

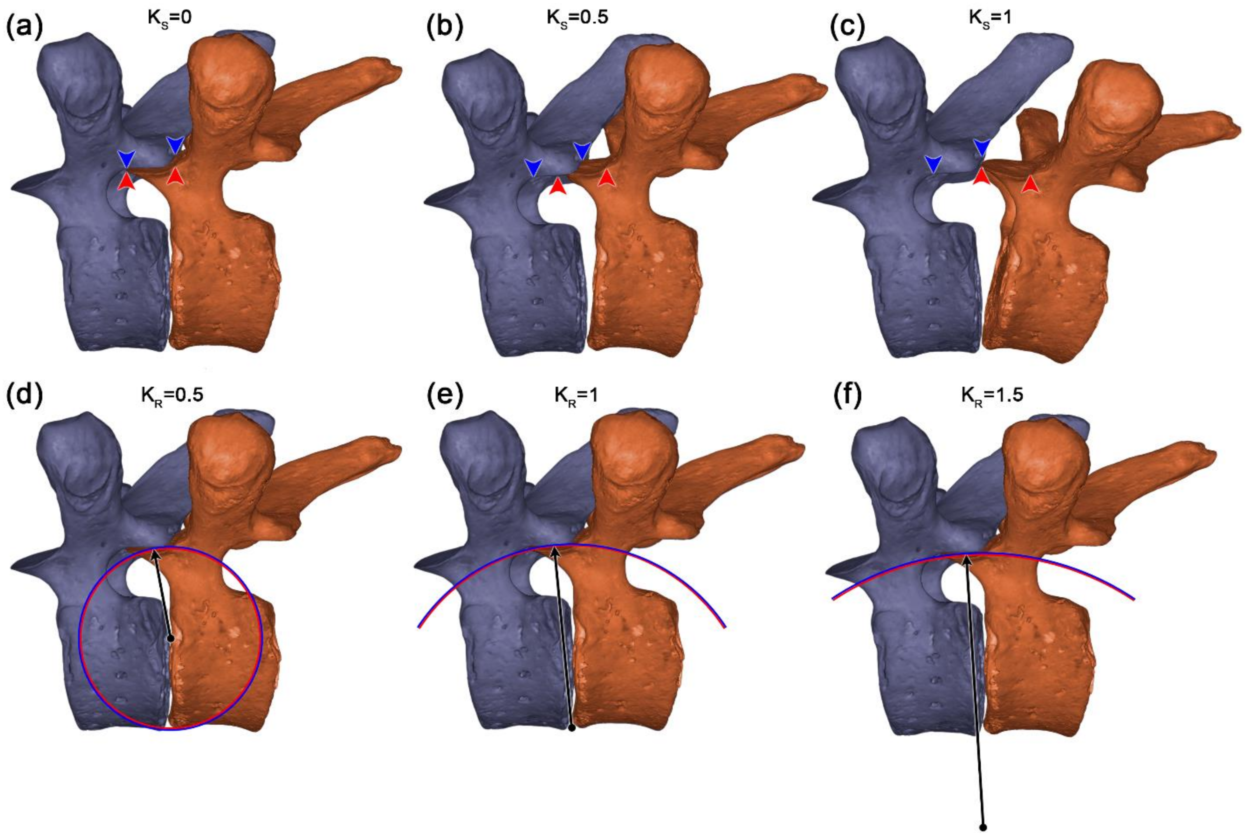

Fig. S2.3 Examples of different values of the formulae coefficients K_S (a-c) and K_R (d-f) applied to the SB mobility of the human T5-T6 joint as seen from the left side. (a-c) represent the relative positions of the facet margins marked with arrowheads. (d-f) represent the center (black dot), the radius (black arrow), and the circular trajectory of motion of the zygapophysial articular facets past each other. Blue corresponds to T5 and red corresponds to T6
