# Supplementary material for: Running, jumping, hunting, and scavenging: Functional analysis of vertebral mobility and backbone properties in carnivorans
Source: J Anat. 2023 Oct 14;244(2):205–31. doi: 10.1111/joa.13955 (PMC10780164; doi:10.1111/joa.13955)
Supplement: Supplementary file 4 — Table S4.1‐S4.4 aROMs in Rf, Tf, and RfL division and lumbar region of the vertebral column in carnivorans [file JOA-244-205-s001.docx]

**Table S4.1** aROMs in Rf division of the vertebral column in carnivorans

| Variable | N | Min (^o^) | Max (^o^) | Mean (^o^) | SD (^o^) | K-S test (P) | Family | | Species | |
| --- | --- | --- | --- | --- | --- | --- | --- | --- | --- | --- |
|  |  |  |  |  |  |  | Min | Max | Min | Max |
| SB mean | 46 | 10.3 | 23.8 | 16.8 | 2.75 | 0.200 | Viverridae | Canidae | *A. melanoleuca* | *N. procyonoides* |
| SB cumul | 46 | 71.8 | 166.3 | 117.3 | 19.95 | 0.200 | Ursidae | Canidae | *A. melanoleuca* | *N. procyonoides* |
| LB mean | 46 | 15.0 | 28.2 | 23.1 | 2.74 | 0.200 | Mustelidae | Canidae | *A. melanoleuca* | *Lycaon pictus* |
| LB cumul | 46 | 105.3 | 197.0 | 160.7 | 20.31 | 0.200 | Mustelidae | Canidae | *A. melanoleuca* | *Lycaon pictus* |
| AR mean | 46 | 5.4 | 9.3 | 7.1 | 1.01 | 0.200 | Canidae | Hyaenidae | *V. vulpes* | *Panthera onca* |
| AR cumul | 46 | 38.0 | 65.3 | 49.2 | 7.22 | 0.200 | Canidae | Hyaenidae | *V. vulpes* | *Panthera onca* |
| Abbreviations: cumul – cumulative | | | | | | | | | | |

**Table S4.2** aROMs in Tf division of the vertebral column in carnivorans

| Variable | N | Min (^o^) | Max (^o^) | Mean (^o^) | SD (^o^) | K-S test (P) | Family | | Species | |
| --- | --- | --- | --- | --- | --- | --- | --- | --- | --- | --- |
|  |  |  |  |  |  |  | Min | Max | Min | Max |
| SB mean | 43 | 4.3 | 11.2 | 6.83 | 1.4 | 0.135 | Hyaenidae | Viverridae | *Cuon alpinus* | *Felis catus* |
| SB cumul | 43 | 34.6 | 89.9 | 58.8 | 12.54 | 0.080 | Hyaenidae | Mustelidae | *Cuon alpinus* | *Felis catus* |
| LB mean | 43 | 9.5 | 15.8 | 11.6 | 1.22 | 0.023 | Hyaenidae | Viverridae | *Hyaena hyaena* | *Felis catus* |
| LB cumul | 43 | 81.1 | 127.4 | 100 | 12.21 | 0.200 | Canidae | Ursidae | *Cuon alpinus* | *Enhydra lutris* |
| AR mean | 43 | 9.15 | 15.3 | 11.2 | 1.19 | 0.040 | Hyaenidae | Viverridae | *Hyaena hyaena* | *Felis catus* |
| AR cumul | 43 | 78.4 | 122.7 | 96.8 | 12.02 | 0.200 | Canidae | Ursidae | *Cuon alpinus* | *Enhydra lutris* |

**Table S4.3** aROMs in lumbar region of the vertebral column in perissodactyls

| Variable | N | Min (^o^) | Max (^o^) | Mean (^o^) | SD (^o^) | K-S test (P) | Family | | Species | |
| --- | --- | --- | --- | --- | --- | --- | --- | --- | --- | --- |
|  |  |  |  |  |  |  | Min | Max | Min | Max |
| SB mean | 48 | 8.2 | 15.6 | 10.91 | 1.6 | 0.200 | Felidae | Viverridae | *Ursus maritimus* | *N. procyonoides* |
| SB cumul | 48 | 47.1 | 109.3 | 70.5 | 13.28 | 0.200 | Hyaenidae | Viverridae | *Cr. crocuta* | *N. procyonoides* |
| LB mean | 48 | 7.8 | 12.6 | 9.78 | 1.11 | 0.200 | Felidae | Hyaenidae | *Cuon alpinus* | *Chr. brachyurus* |
| LB cumul | 48 | 41.5 | 92.0 | 66.61 | 10.35 | 0.200 | Mustelidae | Canidae | *Gulo gulo* | *Chr. brachyurus* |
| AR mean | 48 | 0.7 | 5.8 | 2.88 | 1.28 | 0.200 | Felidae | Ursidae | *L. serval* | *Chr. brachyurus* |
| AR cumul | 48 | 5 | 40.4 | 19.13 | 8.16 | 0.200 | Mustelidae | Ursidae | *L. serval* | *Chr. brachyurus* |

**Table S4.4** aROMs in RfL division of the vertebral column and thoracic RfL joints

| Variable | N | Min (^o^) | Max (^o^) | Mean (^o^) | SD (^o^) | K-S test (p) | Family | | Species | |
| --- | --- | --- | --- | --- | --- | --- | --- | --- | --- | --- |
|  |  |  |  |  |  |  | Min | Max | Min | Max |
| SB mean | 48 | 8.2 | 15.1 | 10.73 | 1.46 | .200 | Felidae | Viverridae | *Ursus maritimus* | *N. procyonoides* |
| SB mean ^a^ | 46 | 7.0 | 15.0 | 10.36 | 1.64 | .193 | Felidae | Viverridae | *Panthera uncia* | *Enhydra lutris* |
| SB cumul | 48 | 65.4 | 140.3 | 99.33 | 16.59 | .200 | Hyaenidae | Viverridae | *Ursus maritimus* | *N. procyonoides* |
| SB cumul ^a^ | 48 | 14.0 | 55.1 | 28.61 | 8.29 | .067 | Ursidae | Mustelidae | *Panthera uncia* | *Meles meles* |
| LB mean | 48 | 8 | 11.9 | 9.74 | 0.98 | .200 | Canidae | Hyaenidae | *Cuon alpinus* | *Chr. brachyurus* |
| LB cumul | 48 | 69.9 | 119.4 | 92.3 | 11.4 | .200 | Hyaenidae | Viverridae | *Hyaena hyaena* | *Chr. brachyurus* |
| AR mean | 48 | 1 | 5.4 | 2.96 | 1.08 | . 200 | Felidae | Ursidae | *Caracal caracal* | *Chr. brachyurus* |
| AR cumul | 48 | 10.9 | 53.9 | 27.93 | 9.99 | .200 | Felidae | Ursidae | *Caracal caracal* | *Chr. brachyurus* |
| a – in the thoracic RfL joints. | | | | | | | | | | |
