# Supplementary figures and images for: Running, jumping, hunting, and scavenging: Functional analysis of vertebral mobility and backbone properties in carnivorans
Source: J Anat. 2023 Oct 14;244(2):205–31. doi: 10.1111/joa.13955 (PMC10780164; doi:10.1111/joa.13955)

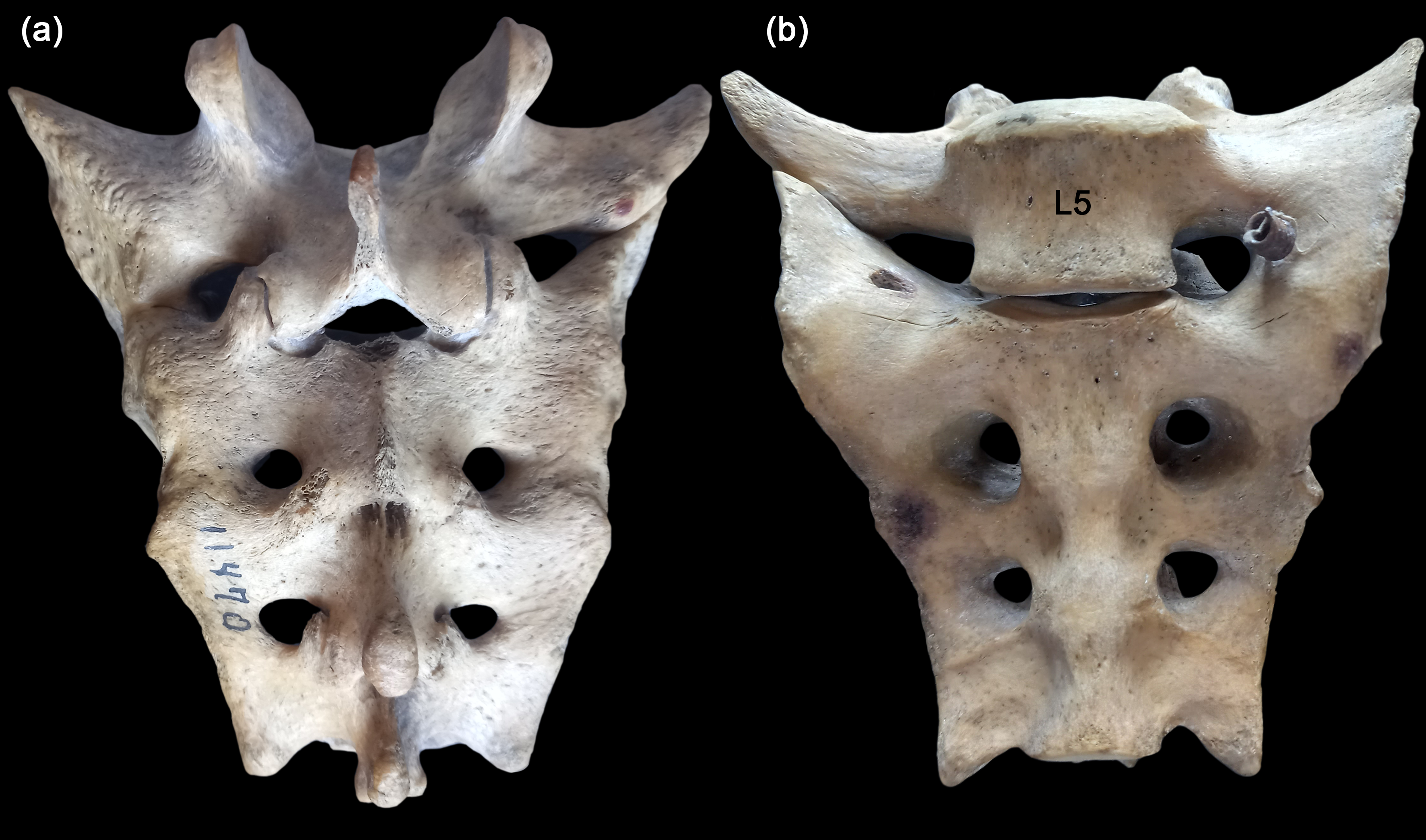

Supplement: Supplementary file 5 — Figure S5 Fusion between L5 and sacrum in Crocuta crocuta (ZIN 11470). Dorsal (a) and ventral (b) view. [file JOA-244-205-s007.png]

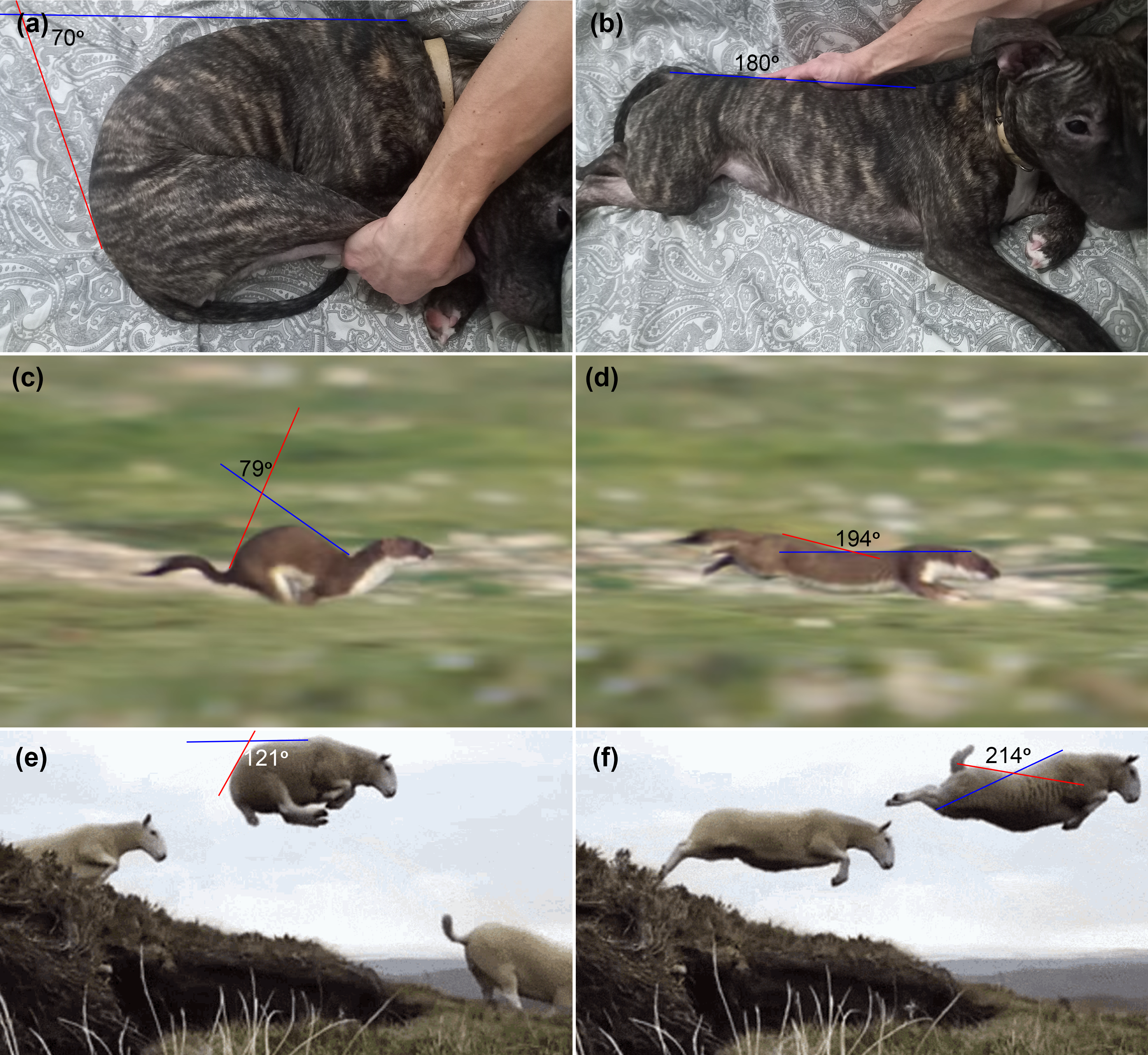

Supplement: Supplementary file 9 — Figure S9 Maximum and minimum sagittal curvature of the lumbosacral part of the backbone in (a‐b) Canis lupus familiaris; (c‐d) the stoat (Mustela erminea) chasing hare (from https://www.youtube.com/watch?v=HNbqvqf3‐14); (e‐f) sheep jumping. The difference between the maximum and minimum angles of the curvature represents SB uROM; it equals 110° in (a‐b), 115° in (c‐d), and 93° in (e‐f). [file JOA-244-205-s004.png]
