# Supplementary material for: Running, jumping, hunting, and scavenging: Functional analysis of vertebral mobility and backbone properties in carnivorans
Source: J Anat. 2023 Oct 14;244(2):205–31. doi: 10.1111/joa.13955 (PMC10780164; doi:10.1111/joa.13955)
Supplement: Supplementary file 7 — Table S7 Phylogenetic PCA analysis description: variables used; eigenvalues and percentage of variance for the pPCA axes; key variables related to the pPC1 and pPC2 axes. [file JOA-244-205-s009.docx]

The first pPCA was performed using nine variables: number of thoracic, lumbar and sacral vertebrae; number of Tf and RfL joints; relative length of the cervical (C/(T+L+S)) and thoracic (T/(T+L)) regions, Tf (Tf/(T+L)) and RfL (RfL/(T+L)) divisions (Table S7.1, Fig 7a). The first principal component (pPC1) explained 54.2% of the variation in studied variables and was mostly related to high numbers of L vertebrae, RfL joints, elongated RfL division, and low numbers of T and S vertebrae, Tf joints, as well as shortened thoracic region and Tf division. The second principal component (pPC2) explained 16.5% of the variation and was mostly related to elongated cervical and thoracic region and low numbers of Tf joints.

**Table S7.1.** Eigenvalues and percentage of variance for the nine pPCA axes.

|  | Eigenvalue | % variance |
| --- | --- | --- |
| Dim.1 | 2.2087874 | 54.21 |
| Dim.2 | 1.2168659 | 16.45 |
| Dim.3 | 1.0085983 | 11.3 |
| Dim.4 | 0.92404072 | 9.49 |
| Dim.5 | 0.59600926 | 3.95 |
| Dim.6 | 0.49030528 | 2.67 |
| Dim.7 | 0.34011354 | 1.29 |
| Dim.8 | 0.214313041 | 0.51 |
| Dim.9 | 0.110182250 | 0.13 |

The second pPCA was performed using four variables: relative length of the cervical (C/(T+L+S)) region, cumulative SB, LB and AR aROM in Rf joints (Table S7.2, Fig 7b). The first principal component (pPC1) explained 62.7% of the variation in studied variables and was mostly related to high SB, LB and AR aROM. The second principal component (pPC2) explained 19.1% of the variation and was mostly related to elongation of cervical region.

**Table S7.2.** Eigenvalues and percentage of variance for the four pPCA axes.

|  | Eigenvalue | % variance |
| --- | --- | --- |
| Dim.1 | 1.5831446 | 62.66 |
| Dim.2 | 0.8748702 | 19.13 |
| Dim.3 | 0.7422823 | 13.77 |
| Dim.4 | 0.4210372 | 4.43 |

The third pPCA was performed using five variables: number of RfL joints, relative length of the RfL (RfL/(T+L)) division, cumulative SB, LB and AR aROM in RfL joints (Table S7.3, Fig 7c). The first principal component (pPC1) explained 48.5% of the variation in studied variables and was mostly related to high numbers of RfL joints, elongated RfL division, and high amplitudes of LB aROM in the RfL division. The second principal component (pPC2) explained 32.4% of the variation and was mostly related to high cumulative SB aROM and low AR aROM in the RfL division.

**Table S7.3.** Eigenvalues and percentage of variance for the five pPCA axes.

|  | Eigenvalue | % variance |
| --- | --- | --- |
| Dim.1 | 1.5573732 | 48.51 |
| Dim.2 | 1.2723252 | 32.38 |
| Dim.3 | 0.7351910 | 10.81 |
| Dim.4 | 0.5523371 | 6.1 |
| Dim.5 | 0.3319567 | 2.2 |

The fourth pPCA was performed using twelve variables: relative length of the cervical (C/(T+L+S)) region, Tf (Tf/(T+L)) and RfL (RfL/(T+L)) divisions; cumulative SB, LB and AR aROM in Rf, Tf and RfL joints (3x3; Table S7.4, Fig 7d). The first principal component (pPC1) explained 33.4% of the variation in studied variables and was mostly related to high amplitudes of cumulative SB, LB and AR aROM in the Tf division, and low cumulative SB and LB aROM in the Rf division, as well as shortened cervical region. The second principal component (pPC2) explained 21.8% of the variation and was mostly related to high AR aROM in the Rf division, elongated cervical region, Tf division and shortened RfL division, as well as low SB aROM in the RfL division.

**Table S7.4.** Eigenvalues and percentage of variance for the twelve pPCA axes.

|  | Eigenvalue | % variance |
| --- | --- | --- |
| Dim.1 | 2.0021113 | 33.4 |
| Dim.2 | 1.6158272 | 21.76 |
| Dim.3 | 1.3360614 | 14.88 |
| Dim.4 | 1.2535452 | 13.09 |
| Dim.5 | 0.82153465 | 5.62 |
| Dim.6 | 0.74340590 | 4.61 |
| Dim.7 | 0.5706334 | 2.71 |
| Dim.8 | 0.47813692 | 1.91 |
| Dim.9 | 0.38321841 | 1.22 |
| Dim.10 | 0.251517027 | 0.53 |
| Dim.11 | 0.138939913 | 0.16 |
| Dim.12 | 0.113960036 | 0.11 |
