# Supplementary material for: Running, jumping, hunting, and scavenging: Functional analysis of vertebral mobility and backbone properties in carnivorans
Source: J Anat. 2023 Oct 14;244(2):205–31. doi: 10.1111/joa.13955 (PMC10780164; doi:10.1111/joa.13955)
Supplement: Supplementary file 8 — Figure S8 Local Moran’s index (Ii) values for each species for the relative neck length (S8.1) and the cumulative LB aROM in the Rf division (S8.2). Red points indicate significant Ii values. [file JOA-244-205-s006.pdf]

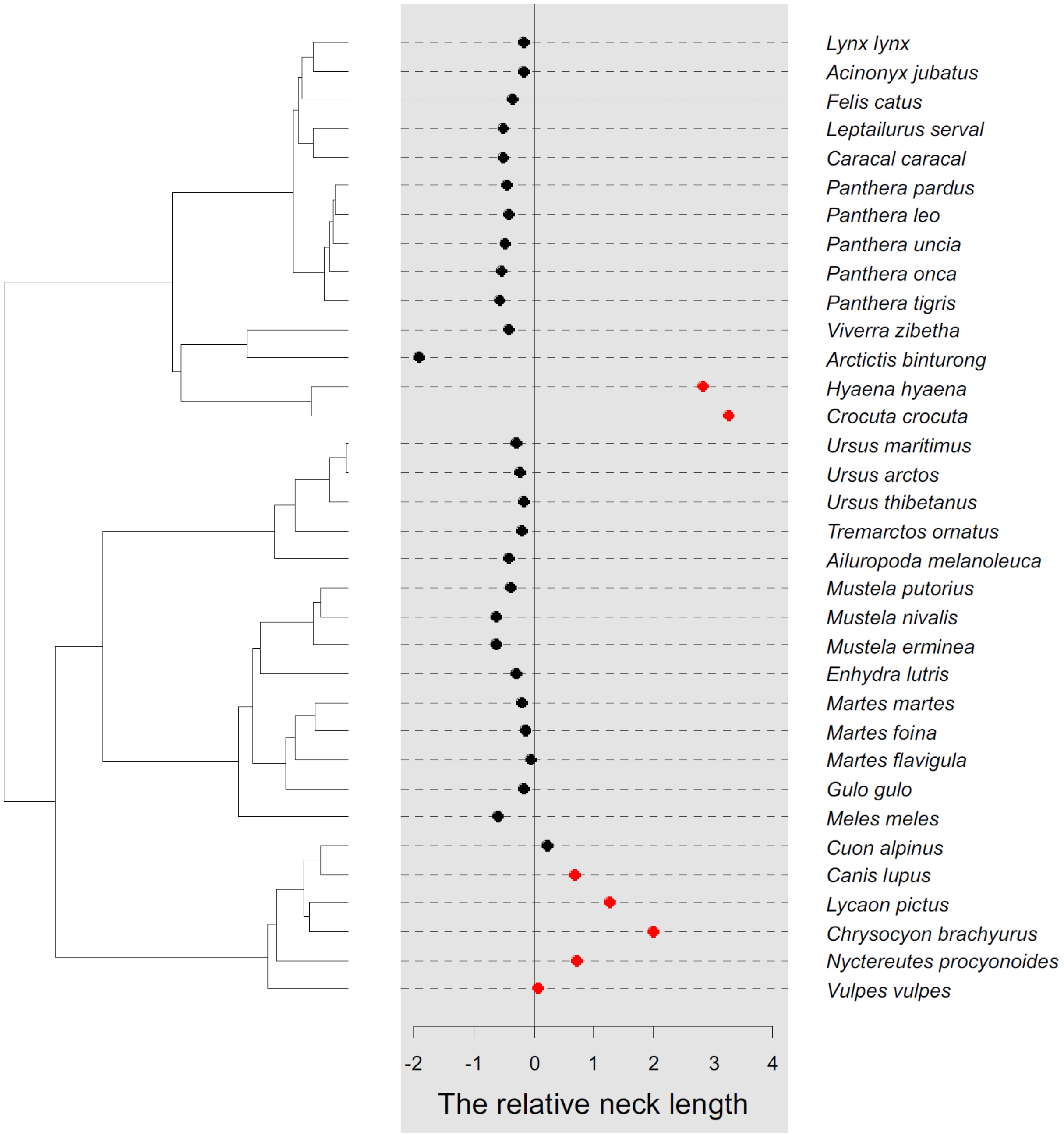

Fig S8.1 Local Moran's index (I_i) values for each species for the relative neck length. Red points indicate significant I_i values.

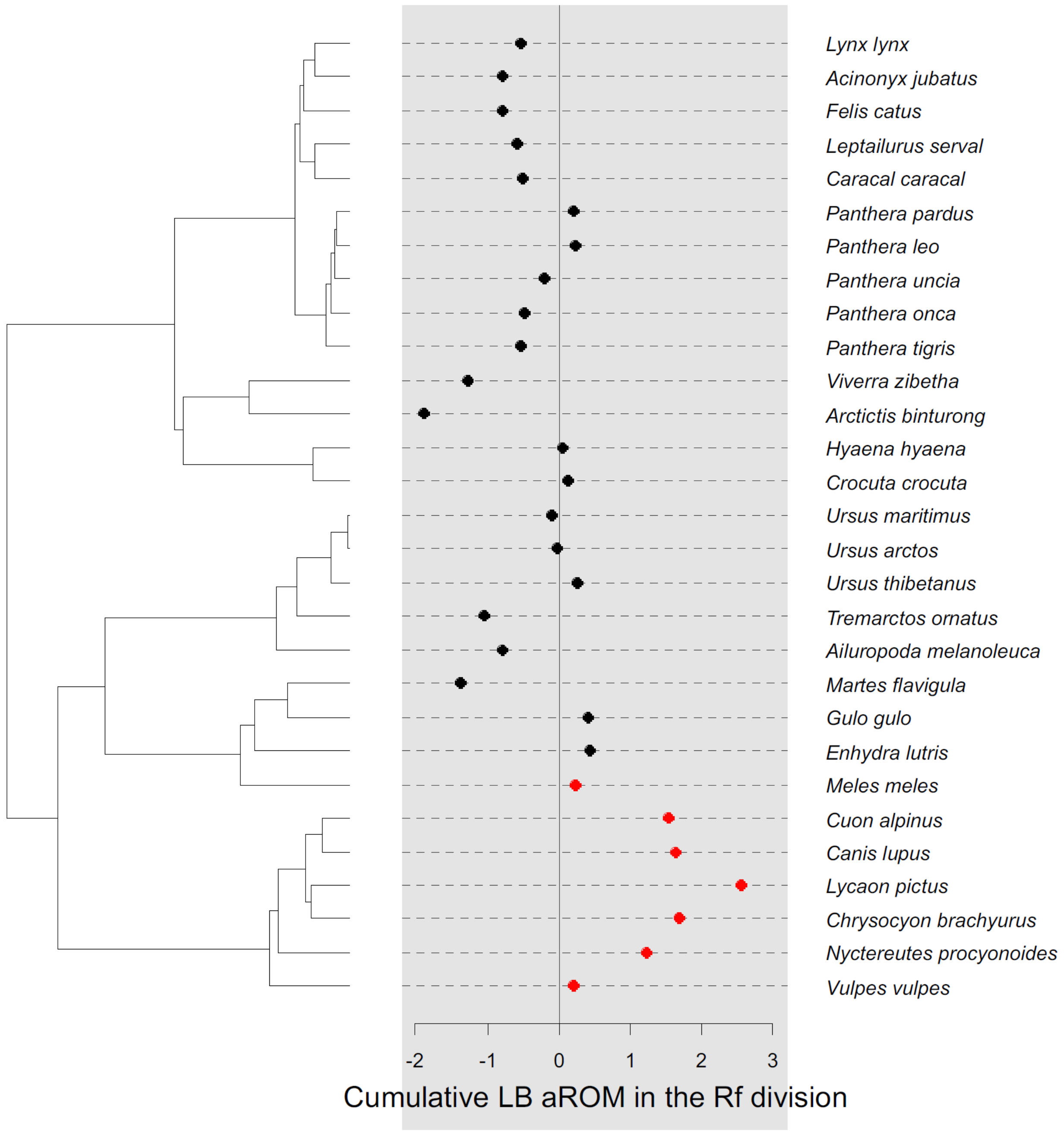

Fig S8.2 Local Moran's index (I_i) values for each species for the cumulative LB aROM in the Rf division. Red points indicate significant I_i values.
